# Meta-analysis of COVID-19 single-cell studies confirms eight key immune responses

**DOI:** 10.1038/s41598-021-00121-z

**Published:** 2021-10-21

**Authors:** Manik Garg, Xu Li, Pablo Moreno, Irene Papatheodorou, Yuelong Shu, Alvis Brazma, Zhichao Miao

**Affiliations:** 1 European Molecular Biology Laboratory, European Bioinformatics Institute (EMBL-EBI), Wellcome Genome Campus, Hinxton, Cambridge, UK; 2 School of Public Health (Shenzhen), Sun Yat-Sen University, Guangzhou, China; 3 Translational Research Institute of Brain and Brain-Like Intelligence and Department of Anesthesiology, Shanghai Fourth People’s Hospital Affiliated to Tongji University School of Medicine, Shanghai, 200081 China

**Keywords:** Infectious diseases, Computational biology and bioinformatics, Biomarkers

## Abstract

Several single-cell RNA sequencing (scRNA-seq) studies analyzing immune response to COVID-19 infection have been recently published. Most of these studies have small sample sizes, which limits the conclusions that can be made with high confidence. By re-analyzing these data in a standardized manner, we validated 8 of the 20 published results across multiple datasets. In particular, we found a consistent decrease in T-cells with increasing COVID-19 infection severity, upregulation of type I Interferon signal pathways, presence of expanded B-cell clones in COVID-19 patients but no consistent trend in T-cell clonal expansion. Overall, our results show that the conclusions drawn from scRNA-seq data analysis of small cohorts of COVID-19 patients need to be treated with some caution.

## Introduction

Coronavirus disease 2019 (COVID-19), the global pandemic caused by severe acute respiratory syndrome coronavirus 2 (SARS-CoV-2), is a recognised major threat to humanity. Thanks to studies from countries around the world, significant progress in the fields of disease diagnosis, treatment, prevention and control of this disease has been made. However, the pathogenesis of SARS-CoV-2 infection and the immunological characteristics associated with the severity of the disease are still unknown.

To understand the pathology and immune response in COVID-19 patients, a number of scRNA-seq experiments have been performed on different cell types obtained from human patients^1–7^. Studies on diseases caused by influenza and other respiratory viruses have shown that the peripheral immune response plays an important role in the defence against the infections and disease progression^1^. In COVID-19 patients, several pathways have been reported to be regulated, including the CCR1 and CCR5 pathways^5^, the HLA class II and type I interferon pathways^2^, the IL1B pathways and interferon-stimulated genes^3^. However, most studies only focus on pathway analysis in certain cell types. Due to the limited patient sample availability, it is difficult to derive statistically reliable trends in the changes of cell subtype proportions over the disease stages in individual studies. It is still unclear to what extent some of the observations can be generalized and which pathways are consistently regulated, therefore, a systematic meta-analysis is needed.

## Results and discussion

**Figure 1:**
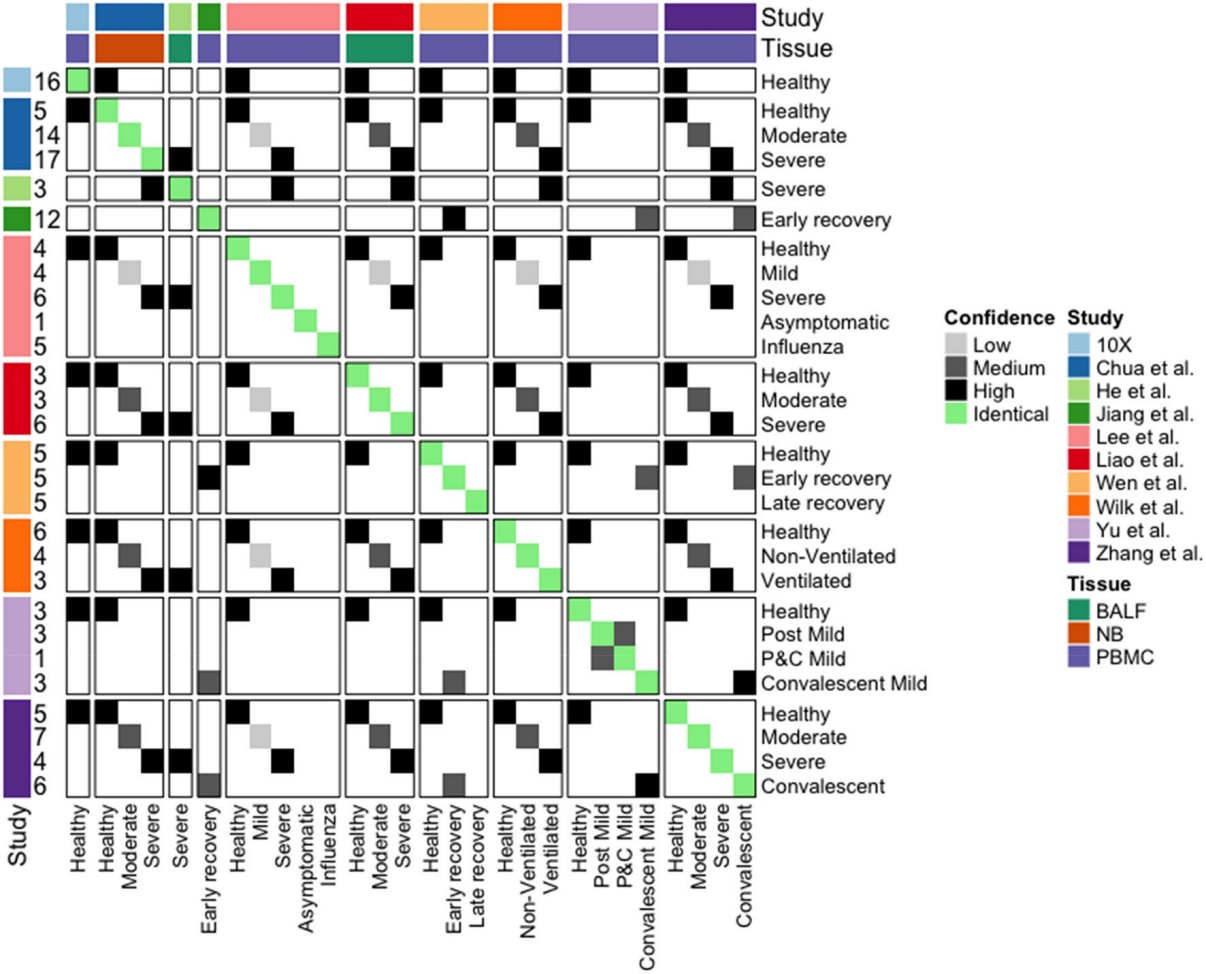
Cross study sample condition comparison. Mapping the conditions in the 10 studies given in Table 1. The number of samples are listed on the left, while the sample tissue types are colored on the top. The similarities between different conditions are colored from white to black (as from no similarity to high similarity). Here, “BALF” denotes samples derived from Bronchoalveolar lavage fluid, “NB” denotes samples derived from nasopharyngeal/bronchial tissue and “PBMC” denotes samples derived from peripheral blood mononuclear cells.

**Figure 2:**
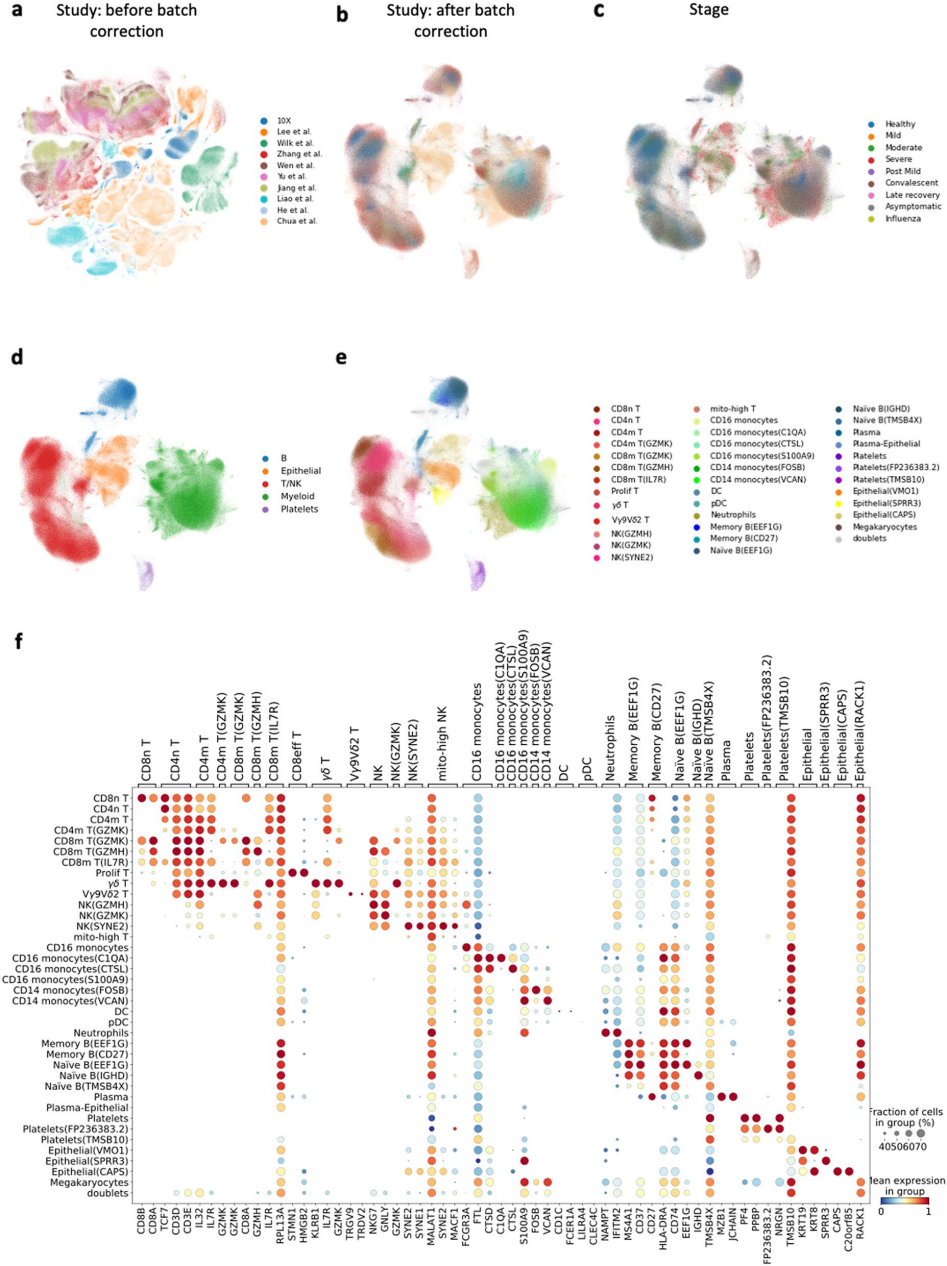
Meta-analysis identifies common and tissue-specific immune cell types. (**a**) The UMAP representation of the cells before batch correction, in which the cells are colored by study; (**b**) the UMAP representation of the cells after batch correction; (**c**) the UMAP representation colored by the disease stage when the sample were taken from the patients; (**d**) the UMAP colored by the main cell types; (**e**) the description of the cell populations in detail; (**f**) the marker genes for discriminating the cell subpopulations.

For the presented meta-analysis, all COVID-19 scRNA-seq available by 9th Oct 2020 were considered (Supplementary Table S1), while the 9 datasets we could get access to and were extracted from peripheral mononuclear cells (PBMC) or bronchoalveolar lavage fluid (BALF) or nasopharyngeal/bronchial (NB) tissues were included in this study. These 9 scRNA-seq datasets are summarised in Table 1, which also gives each dataset a name and a healthy control dataset. In total, the studies comprise 159 samples and 862,354 cells across 9 different disease conditions. We map the disease stages to standardised terms Healthy, Mild, Moderate, Severe, Post Mild, Convalescent, Late recovery, Asymptomatic and Influenza (Supplementary Table S2). Although such mapping may introduce a certain level of noise, it is essential for meta-analysis (Fig. 1), and we have taken care to consider the detailed descriptions in the respective publications (see Supplementary Data 1). Visualisation of the combined data (Fig. 2a) reveals strong batch-effects, however after the data integration with Harmony^8^, the cells from different studies largely clustered by the underlying biology (Fig. 2b), and moreover, with minor exceptions, the healthy samples are well-mixed (Fig. 2c).

**Table 1:** The scRNA-seq studies of SARS-CoV-2 infected patients samples included in this meta-analysis.

Study	Accession	Tissue	Technique	5′ or 3′	Samples	Cells	TCR/BCR
Wen et al.^3^	PRJCA002413	PBMC	10×	5′	15	119,448	TCR + BCR
Zhang et al.^4^	PRJCA002564	PBMC	10×	5′	22	140,588	TCR + BCR
Lee et al.^1^	GSE149689	PBMC	10×	3′	20	58,022	–
Yu et al.^6^	PRJCA002579	PBMC	10×	5′	10	91,742	–
Jiang et al.^9^	NA	PBMC	10×	NA	12	93,804	–
Wilk et al.^2^	GSE150728	PBMC	Seq-Well	NA	13	67,923	–
Liao et al.^10^	GSE145926	BALF	10×	3′	12	63,103	TCR
He et al.^7^	GSE147143	BALF	10×	3′	3	10,927	–
Chua et al.^5^	EGAS00001004481	NB	10×	3′	36	156,025	–
10×	NA	PBMC	10×	3′	16	60,772	–

The 10 datasets used in this meta-analysis study include 6 datasets (Lee, Wilk, Zhang, Wen, Yu, Jiang) of Peripheral blood mononuclear cells (PBMCs), 2 datasets (Liao and He datasets) of Bronchoalveolar lavage fluid (BALF), 1 dataset (Chua dataset) of nasopharyngeal and bronchial (NB) samples and 10× healthy control dataset of PBMCs. TCR: T-cell receptor repertoire sequencing data; BCR: B-cell receptor repertoire sequencing data; NA: Not applicable.

Similar to previous publications^3,10^, the cells are classified into 5 major cell populations (Fig. 2d): Lymphoid cells, Myeloid cells, B cells, Epithelial cells and Platelets (marker gene expressions are shown in Supplementary Fig. S1, while previously reported marker gene lists are summarized in Supplementary Data 2. Cells in each population are then further divided into subpopulations based on the expression of marker genes and logistic regression reference-based annotation methods (see “Methods” section).

By integrating the datasets, we are able to refine the annotation of cell subpopulations. For example, monocyte cells have been discussed in a previous publication^11^, however we are able to refine this type to include four CD16^+^ monocytes subpopulations and two CD14^+^ monocytes subpopulations (Supplementary Fig. S5). These cell clusters correlate well with the cell populations reported by Schulte-Schrepping et al.^11^. Also, neutrophils were not annotated in all the reported datasets^4^. However, after annotating neutrophils according to the canonical markers (*FCGR3B* and *CXCR2*), Supplementary Fig. S6, we find that they exist in all disease samples (but considerably less in the healthy controls). The resulting cell clusters were further refined using SCCAF^12^ all the 5 major cell populations achieved self-projection accuracies above 92% in the Harmony latent space (Supplementary Table S3). The final result includes 37 cell subpopulations, excluding doublets (Fig. 2e, Supplementary Fig. S7). A more detailed description of these subpopulations is given in the Supplementary Material Note 1.

After obtaining consistent cell-type annotations across the datasets, we moved on to validating the published results. Specifically, we analysed (1) the cell-type proportion changes captured in different experiments to infer the immune response upon SARS-CoV-2 infection; (2) the gene expression regulation and pathway activation in COVID-19 patients; (3) the clonal expansion in T cells and B cells. We could reproduce 16 out of 20 (80%) published results in the original datasets, while only 8 out of 20 (40%) results across the datasets (Table 2). More detail and possible explanations for the limited reproducibility in each case are given in Supplementary Table S4.

**Table 2:** Summary of reproducible results across multiple single-cell COVID-19 studies analyzed in this meta-analysis.

Study	Key finding(s) in COVID-19 patients compared to healthy controls	Reproduced in the original dataset	Reproduced in all relevant datasets
Wen et al.^3^	Decreased CD4^+^ and CD8^+^ T-cells (data not shown)	✓	✓
	Decreased T-cell clonal expansion in convalescent patients compared to healthy controls (Supp Fig. S15c)	×	×
	Increased CD14^+^ monocytes (Supp Figs. S9g,h)	✓	×
	Increased B-cell clonal expansion (Supp Fig. S17b)	✓	✓
	Increased Plasma cells (Fig. 3f, Supp Fig. S9h)	✓	✓
	Decreased naïve B-cells (Supp Fig. S9c,d)	×	×
Zhang et al.^4^	IFN-ɑ response upregulation (Supp Figs. S10d,e, S11)	✓	✓
	Increased T-cell clonal expansion (Supp Fig. S15c)	✓	×
	Increased CD8eff T-cell clonal expansion (Supp Figs. S15a,b, S16b)	✓	✓
	Increased Plasma cells (Fig. 3f)	✓	✓
	Decreased memory B-cells (Supp Fig. S9b)	✓	×
Lee et al.^1^	TNF/IL-1β driven inflammatory response upregulation (data not shown)	×	×
	Co-existence of type I IFN response with TNF/IL-1β-driven inflammation in severe COVID-19 patients (data not shown)	✓	×
Wilk et al.^2^	Developing neutrophil population from plasmablasts in severe COVID-19 patients (Supp Figs. S13, S14)	✓	×
	HLA-class II downregulation in CD14^+^ monocytes (Supp Fig. S10f)	✓	✓
	Heterogenous ISG module upregulation in CD14^+^ monocytes (Supp Fig. S10g)	✓	×
	Presence of type I IFN driven inflammatory signatures in CD14^+^ monocytes (Supp Fig. S10d,e)	✓	✓
	Lack of substantial expression of pro-inflammatory cytokine genes (*TNF, IL6, IL1B, CCL3, CCL4 or CXCL2*) in CD14^+^ monocytes (data not shown)	✓	×
Liao et al.^10^	Increased CD8^+^ T-cell clonal expansion in moderate COVID-19 patients compared to severe (Supp Figs. S15d, S16b)	×	×
Chua et al.^5^	Activated macrophages expressing inflammatory chemokines including *CCL2, CCL3, CCL20, CXCL1, CXCL3, CXCL10, IL8, IL1B* and *TNF* in severe patients (data not shown)	✓	×

**Figure 3:**
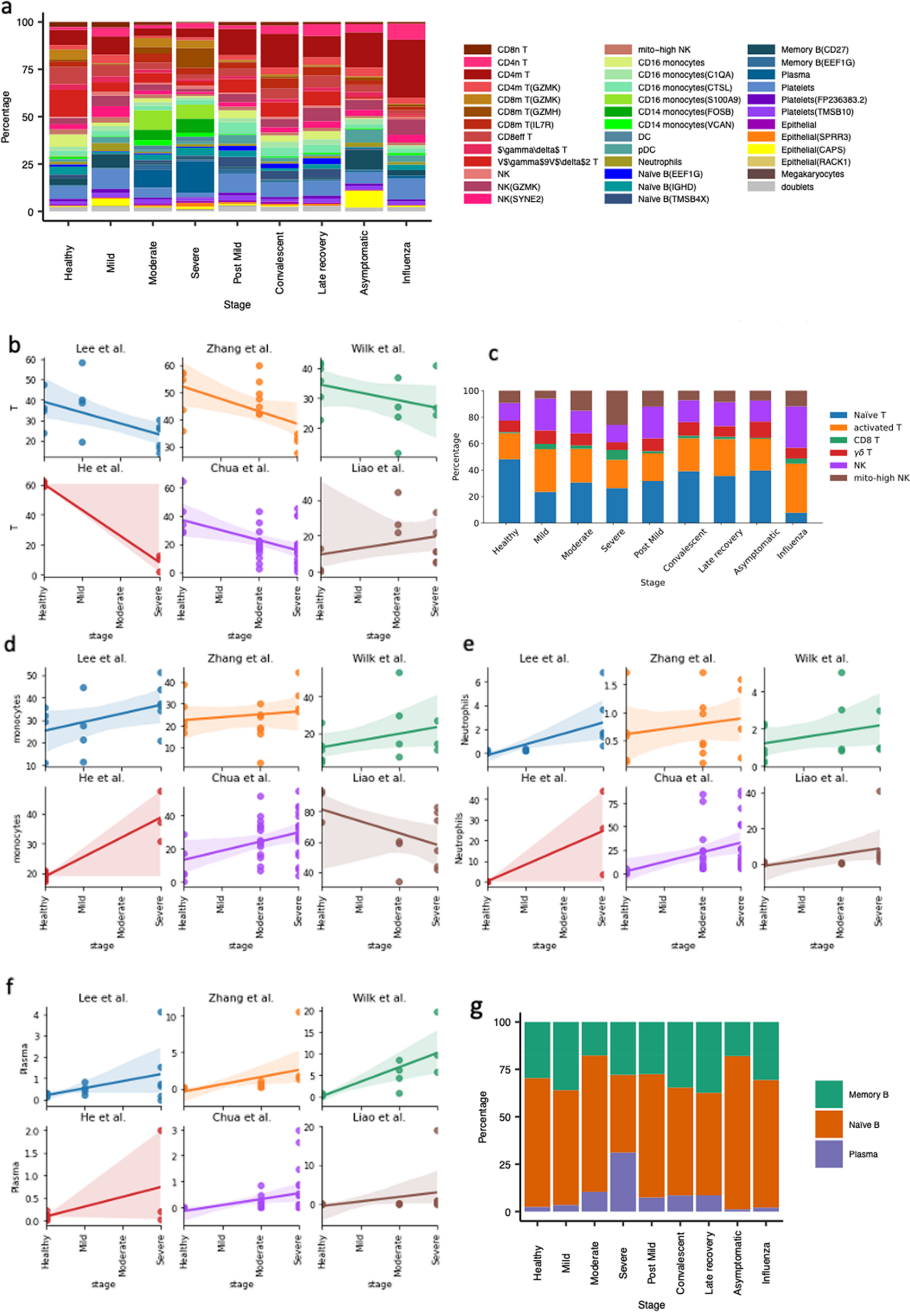
Cell type proportion change upon SARS-CoV-2 infection revealed by multiple studies. (**a**) The general distribution of the cell subpopulations at different disease stages; (**b,d–f**) The cell proportion changes upon disease stages for T cells, monocytes, neutrophils and plasma cells, respectively, in different studies. (**c–g**) The general distribution of T-cells/Natural killer (NK) cells and B-cells, respectively, across stages.

We suspect the main reasons for non-reproducibility in the study’s own datasets to be the heterogeneity in data processing and analyzing pipelines along with differences in cell-annotation. Non-reproducibility in other datasets could be due to heterogeneity among datasets collected from different small cohorts of COVID-19 patients along with the differences in stage mapping. We also called a result “reproducible” only if it was observed in all the datasets considered for comparison, even though the dataset was collected from a different tissue. This stringency might have also reduced the number of results we considered as reproducible. For more detailed explanations, please refer to the Supplementary Table S4. TNF: tumor necrosis factor; IL: interleukin; HLA: human leukocyte antigen; CD8eff T-cell: CD8^+^ effector T-cell; Supp: Supplementary; ISG: Interferon stimulated genes.

In addition, Fig. 3a gives an overview of the cell populations’ proportions across all the stages (PBMC and BALF specific information given in Supplementary Fig. S8a,b, respectively). We observe that 4 datasets (Lee, Zhang, Wilk and Chua) show a similar proportion of T cells in healthy donors, which is around 30–40%, while the 10× healthy PBMC reference shows ~ 60% T cells and the Liao dataset of BALF shows only ~ 10% T cells (Fig. 3b). Due to the lack of bronchoalveolar lavage fluid (BALF)-derived healthy samples in the He dataset, we cannot confirm whether this is expected in healthy BALF samples or if it corresponds to the general individual-specific variability, for instance, as explored by Wong et al.^13^. However, we can still find a decrease of T cell proportion from moderate to severe stages in the Liao dataset. An overview could be found in Fig. 3c and Supplementary Fig. S8c,d.

The percentages of monocytes and neutrophils relatively increase from healthy to severe stages. Similar to the T cell proportion distribution, the Lee, Zhang, Wilk, Chua and 10× datasets show ~ 20% monocytes in the healthy samples (Fig. 3d). In the Liao data this percentage is around 80% and it still shows an increase in monocytes from moderate to severe stage. If we use 20% as a reference value for monocytes in healthy controls, monocytes further increase from healthy to severe patients in the Liao dataset. For neutrophils, the 6 datasets consistently illustrate an increasing trend from healthy to severe, though the levels of increase can vary (Fig. 3e). In the He and Chua dataset, some of the samples include 40–70% neutrophils. As mentioned in Table 2 (and Supplementary Table S4), we observed an increase in the proportion of plasma cells from healthy to severe patients (Fig. 3f–g, Supplementary Fig. S8e,f, S9h) with no clear pattern in memory B and näive B cells (Fig. 3g, Supplementary Figs. S8e,f, S9b,c).

Although dendritic cells take up only a small portion of the population (~ 2% in healthy PBMCs), they decrease over the disease stages in Lee, Zhang, Chua and Liao datasets (Supplementary Fig. S9e). Plasmacytoid dendritic cells are even fewer than dendritic cells, the Zhang and Wilk datasets show clear increasing trends over the disease stages (Supplementary Fig. S9f). This conclusion however cannot be derived from other datasets (Supplementary Fig. S9f).

Furthermore, while looking at the response of gene regulatory pathways to COVID-19 infection, we observed that the log fold changes of the differentially expressed (DE) genes (False Detection Rate < 0.01) between severe and healthy samples correlate with that between moderate and healthy. This indicates that the genes upregulated and downregulated in moderate and severe samples compared to the healthy controls correlate (Supplementary Fig. S10b,c). This correlation happens in most of the cell types in all studies (including Zhang, Wilk, Liao and Chua). We also found that the correlation between mild/healthy (Lee dataset) and severe/healthy is lower than moderate/healthy and severe/healthy; nevertheless, this correlation too is positive in all the cell types.

We also identified the genes upregulated in both moderate and severe samples for each study and checked the overlaps between different studies. In CD14^+^ monocytes, the pathway analysis of 153 consistently upregulated genes across all the three studies (Supplementary Fig. S10d) show overrepresentation in immune response, response to virus and type I interferon signalling pathway (Supplementary Fig. S10e). Although the upregulation of type I interferon signalling pathway in monocytes has been reported by Wilk et al.^2^, the upregulation is observed mostly in CD14^+^ monocytes and not in CD16^+^ monocytes. We find the upregulation of type I interferon signalling pathway (specifically, IFIT, IFI, ISG and OAS genes) to be consistent for many of the cell types, including T cells (CD4^+^ T, CD8^+^ T and γδ T), NK cells, DC, pDC, B cells, Plasma cells and Neutrophils (Supplementary Fig. S11, Supplementary Material Note 2.1). Similarly, genes related to SRP-dependent co-translational protein targeting the membrane as well as the mitochondrial proteins are consistently downregulated in immune cell types. In NK and γδ T cells, similar sets of genes are downregulated, including mitochondrial genes, *KLRB1* and *UBA52* in all the three datasets (Zhang, Wilk, Liao) (Supplementary Material Notes 2.2, 2.3).

Next, we analyzed the T-cell receptor (TCR) repertoire data from Wen et al.^3^, Zhang et al.^4^ and Liao et al.^10^ where the samples from the first two studies were derived from PBMC, while those from the third one were derived from BALF. Among these, the conditions healthy and convalescent were present in both the PBMC datasets, while the moderate and severe stages were present in the Zhang^4^ and Liao^10^. We first pooled T-cell and NK cell data from all the studies together and visualized them using UMAP (Supplementary Fig. S6: “Lymphoid cells”, Supplementary Fig. S15a). As expected, NK-cells mostly had no corresponding TCR data. For the cells with TCR data, we observed clonal expansion of CD8m T-cells, particularly the subpopulations CD8m T(GZMH) and CD8m T(GZMK) and Vγ9Vẟ2 T-cells (Supplementary Fig. S15a), however, the expansion did not appear to be stage-specific (Supplementary Fig. S15a). We also observed an over-representation of CD8^+^ effector T-cells in severe patients (Supplementary Fig. S15a). These observations were confirmed in the bar plots where samples from all the datasets at a specific stage were pooled and normalized together according to the total number of cells at each stage (Supplementary Fig. S15b). This is also consistent with Zhang et al.^14^. To further check if these observations were consistent across studies, we analyzed the overall clonal expansion trend of T-cells. Contrary to previously published results^3,4^, we didn’t observe a consistent trend of increased clonal expansion of T-cells in COVID-19 patients compared to healthy controls (Supplementary Fig. S15c, Table 2, Supplementary Table S4). Further details of the clonal expansion status of specific T-cell subpopulations are given in Supplementary Material Note 3.1.

Upon visualizing the B-cell receptor (BCR) repertoire data from Wen et al.^3^ and Zhang et al.^4^ using the UMAP representation, we noticed the presence of clonally expanded plasma cells, mostly in COVID-19 patients (Supplementary Fig. S17a). This observation was confirmed when cells from all the studies at each stage were taken together and normalized by the total number of cells present at each stage (Supplementary Fig. S18a). Increased clonal expansion of Naïve B(EEF1G) and Memory B(EEF1G) cell subpopulations was also observed in severe COVID-19 patients compared to other B-cell subpopulations in severe patients (Supplementary Fig. S18a). We further analyzed whether the previously reported observations^3,4^ of increased B-cell clonal expansion in COVID-19 patients compared to healthy controls can be confirmed across studies and found that this is indeed consistent (Supplementary Fig. S17a). More details about the clonal expansion status of B-cell subpopulations across multiple studies is given in Supplementary Material Note 3.2.

Finally, we checked whether we could validate the results of the recently published Ren et al.^15^ in these 9 datasets and found that 3 out of 10 observations are indeed reproducible in most of the datasets, including the presence of cytokine storm by CD14+ monocytes (Supplementary Table S4). Since we did not re-analyze the Ren et al.^15^ dataset in a standardized manner with others, there is also a difference in the cell-type annotations complicating any one-to-one comparisons to Ren et al.^15^ dataset (Supplementary Fig. S19b).

## Conclusions

Although our meta-analysis is able to address some issues arising from limited sample size and lack of standardization in data collection, pre-processing, cell-type annotation and analysis, it has obvious limitations. A part of the reason why we were not always able to confirm all the published conclusions in the author's own dataset may be due to the remapping of the scRNA-seq reads to different genome builds and using different methods. For instance, we applied an additional cut-off of 100 cells per sample for each T-cell subpopulation, which was different in the analysis of Liao et al.^10^ (Supplementary Fig. S16a). Nevertheless, the discrepancies that we found, still indicate that the conclusions from early scRNA-seq studies of COVID-19 patients may not always be robust and need to be validated before fully relied upon. The explanation of even larger difficulties in validating most conclusions across datasets, may be the result of inconsistent mapping between the disease stages in different studies and differences in protocols used. The samples used in different studies were collected from patients at different stages and were not annotated on a standardized scale (e.g. the WHO scale^16^). Therefore, we had to make various assumptions, in particular, we assumed that the healthy, severe, early recovery (convalescent) and asymptomatic stages are comparable. Also, four datasets had their sampling day after symptom onset information missing in the source publications^1,3,6,7^ (Supplementary Data 1) which could have contributed to additional heterogeneity between samples across datasets. Other limitations are mentioned in Supplementary Material Note 4. Overall, our results show that the conclusions drawn from scRNA-seq data analysis of small cohorts need to be treated with some caution.

## Methods

### Data collection

#### 10× chromium healthy control

The 10× Chromium data for healthy PBMCs was downloaded from the 10× genomics website [https://support.10xgenomics.com/single-cell-gene-expression/datasets/3.1.0/5k_pbmc_NGSC3_aggr]. This dataset includes samples from 8 Chromium Connect channels and 8 manual channels.

#### Wen dataset

The raw fastq files for the Wen dataset^3^ were downloaded from the GSA (Genome Sequence Archive) database^17,18^ under the accession number PRJCA002413, including all the files for scRNA-seq, TCR-seq and BCR-seq.

The fastq files were aligned to the human genome (GRCh38 version 3.0.0, including 33,538 genes), which was downloaded from the 10× Chromium website [https://support.10xgenomics.com/single-cell-gene-expression/software/downloads/latest], using Cell Ranger (3.1.0). TCR and BCR results were aligned to the human vdj reference (version 4.0.0) from 10× Chromium website using Cell Ranger (3.1.0).

#### Liao dataset

For the Liao dataset^10^, the Cell Ranger mapped results together with the TCR results were downloaded from GEO^19^ under the accession number GSE145926. The downloaded scRNA-seq data includes 33,538 features, which is the same as the reference genome used in the above mentioned datasets. The cell type annotation of the dataset was downloaded from GitHub [https://raw.githubusercontent.com/zhangzlab/covid_balf/master/all.cell.annotation.meta.txt]. The unannotated cells were considered as low quality and were excluded from analysis.

#### Lee dataset

The Lee dataset^1^ was downloaded from GEO under the accession number GSE149689. The expression matrix also includes 33,538 features, which is the same as the reference genome used in the above-mentioned datasets.

#### Yu dataset

We downloaded the raw fastq files for the Yu dataset^6^ from the GSA (Genome Sequence Archive) database under the accession number PRJCA002579. Reads mapping was performed in the same way as on the Wen dataset.

#### Jiang dataset

The mapped results for the Jiang dataset^9^ was downloaded from Fig.share [https://figshare.com/articles/dataset/Single_cell_and_immune_repertoire_profiling_of_COVID-19_patients_reveal_novel_therapeutic_candidates/12115095]. The expression matrix also includes 33,538 features, which is the same as the reference genome used in the above mentioned datasets.

#### Wilk dataset

We downloaded the raw fastq files of the Wilk dataset^2^ from the ENA database^20^ under the accession number PRJNA633393. According to Wilk et al., dropEst, samtools and STAR were used for the reads mapping. Human genome GRCh38 version 3.0.0 (the same as the Wen dataset reference) was used as the reference genome for reads mapping. The resulting expression matrix includes 33,538 features for 150,245 cells.

#### Zhang dataset

We downloaded the raw fastq files of the Zhang dataset^4^ from the GSA (Genome Sequence Archive) database under the accession number HRA000150. Reads mapping was performed in the same way as on the Wen dataset.

#### He dataset

We downloaded the mapped results of the He dataset^7^ from GEO under the accession number GSE147143. The expression matrix includes 33,578 features, 24,020 of which are overlapped with the features in the reference genome used in the Wen dataset.

#### Chua dataset

The mapped results of the Chua dataset^5^ were downloaded from Fig.share [https://doi.org/10.6084/m9.figshare.12436517] as mentioned in the publication. The expression matrix includes 26,924 features, 18,410 of which are overlapped with the features in the reference genome used in the Wen dataset. The ‘celltype’ column in the dataset was used as cell type annotation.

### Data processing

#### Quality control

All the datasets include 33,538 features except the He and Chua datasets. For the He and Chua datasets, only the gene symbols that overlap with the other datasets are considered. All the datasets are combined together for quality control and downstream analyses. We filtered the cells with higher than 15% of mitochondrial contents. We also excluded cells of fewer than 500 UMIs or fewer than 200 features. Features expressed in fewer than 3 cells were removed in the analysis. We used Scrublet (version 0.2.1)^21^ to determine the doublets in the datasets. As Scrublet was designed to deal with 10× data, the result on the Wilk dataset was not used. Cell clusters represented by doublets were removed from the analysis (Supplementary Fig. S20). The cell numbers after quality control were listed in Table 1.

#### Data integration

SCANPY^22^ workflow was used to analyze the data. The following steps were performed: data normalization, log-transformation, highly variable genes selection using the ‘cellranger’ flavor and principal component analysis. Then, we used Harmony (version 0.0.5)^8^ to integrate data from different samples. UMAP^23^ and Louvain^24^ clustering were calculated according to the Harmony corrected latent space.

#### Cell type annotation

For the eight datasets of the same reference genome (Wen, Liao, Lee, Yu and Jiang datasets) we combined the dataset to annotate all the cell types. To obtain good cellular annotations, we used two approaches: a machine learning-based cell type annotation approach using a reference dataset and manual annotation with previously reported marker genes (as summarized in Supplementary Data 2). Specifically, we first used logistic regression in SCCAF^12^ to train a machine learning model using the possible cell type labels according to a reference dataset (the Wilk dataset)^2^. Then, each Louvain cluster was assigned to a cell type label according to the machine learning-annotated label. For the Wilk dataset and Chua dataset, we used the cell type annotations adopted from their publications. For the He dataset, we annotated the cell types according to the marker gene expression. We then checked all these marker genes’ expression in the integrated dataset to assure our annotation (Fig. 2f). According to the results, our annotations are highly consistent among the datasets (Supplementary Data 2, Fig. 2b). They are also comparable to the known annotations reported in previous papers (e.g., the Wilk dataset) (Supplementary Figs. S1–S6). For the downstream analysis, we first excluded the platelets (*PPBP, PF4*) and epithelial cells (*TPPP3, KRT18*) clusters. According to the published marker genes (Supplementary Data 2), we divided the remaining data into three populations: Lymphoid cells (*CD3D, CD3E, NKG7, NCAM1*), Myeloid cells (*CD68, CD14, FCGR3A, CD1C*) and B cells (*CD19, MS4A1, CD79A, MZB1*).

#### Differential expression and Gene Ontology analysis

To account for the technical effects such as number of cells or sequencing depth, the hurdle model in MAST^25^ was used to model the differential expression of the cells. Only the genes with a false detection rate (FDR) lower than 0.01 was used for volcano plot and later gene ontology analysis.

In the MAST results, the genes with a log fold change value greater than 0 are considered as up-regulated genes, while the rest are the down-regulated genes. And the up-regulated genes in a cell cluster are used for gene ontology analysis. The python package of GProfiler^26^ was used to understand the pathway regulation. The gene module scores for HLA class II and ISG signature were calculated for each individual cell using sc.tl.score_genes function of scanpy^22^ v1.5.1.

#### TCR/BCR analysis

The TCR and BCR V(D)J data from the studies Wen et al.^3^, Zhang et al.^4^ and Liao et al.^10^ (TCR only) were analyzed separately using the python library pyvdj^27^ v0.1.2. For TCR V(D)J data, only cells with at least one productive TRA and at least one productive TRB chain were considered for analysis. Similarly, for BCR V(D)J data, only cells with at least one productive IGH and at least one productive IGL or IGK chain were considered for analysis. The UMAP plots were generated using scanpy^22^ v1.5.1. The boxplots and barplots were generated using R-package ggplot2^28^ v3.3.2, ggpubr^29^ v0.4.0.999, rstatix^29,30^ v0.6.0.999 and tidyverse^31^ v1.3.0.

## Supplementary Information

Supplementary Information 1.

Supplementary Information 2.

Supplementary Information 3.

## Data Availability

For the studies included in the current manuscript, the raw sequencing data are available in the European Genome-phenome Archive (EGAS00001004481 for Chua et al.^5^), GEO (GSE149689 for Lee et al.^1^, GSE150728 for Wilk et al., GSE145926 for Liao et al.^10^) and Genome Sequence Archive (PRJCA002413 for Wen et al.^3^, PRJCA002564 for Zhang et al.^4^, PRJCA002579 for Yu et al.^6^, GSE147143 for He et al.^7^). Details are listed in Table 1. All source data will be provided with this paper. The merged dataset can be visualised interactively through cellxgene in the Human Cell Atlas Galaxy. EU instance^32^, following instructions in the Supplementary Material Note 5.

## Code availability

The code for reproducing the analysis results and Figures will be made available at the Github repository https://github.com/Manikgarg/COVID-19.
