# Supplementary material for: Meta-analysis of COVID-19 single-cell studies confirms eight key immune responses
Source: Sci Rep. 2021 Oct 21;11:20833. doi: 10.1038/s41598-021-00121-z (PMC8531356; doi:10.1038/s41598-021-00121-z)
Supplement: Supplementary file 3 — Supplementary Information 3. [file 41598_2021_121_MOESM3_ESM.docx]

# Supplementary Tables

**Supplementary Table 1. Summary of datasets considered for meta-analysis and reason for non-inclusion.** PBMC: Peripheral blood mononuclear cells; BALF: Bronchoalveolar lavage fluid; NB: Nasopharyngeal/Bronchial

| S.No. | Study | Accession | Tissue | Reference | Included | Reason for non-inclusion (if applicable) |
| --- | --- | --- | --- | --- | --- | --- |
| 1 | Liao et al. | GSE145926 | BALF | [Nature Medicine 26, 842–844(2020)](https://www.nature.com/articles/s41591-020-0901-9) | Yes |  |
| 2 | He et al. | GSE147143 | BALF | [Protein & Cell](https://link.springer.com/article/10.1007/s13238-020-00752-4) | Yes |  |
| 3 | Lee et al. | GSE149689 | PBMC | [Science Immunology 5, 49, eabd1554](https://immunology.sciencemag.org/content/5/49/eabd1554) | Yes |  |
| 4 | Wilk et al. | GSE150728 | PBMC | [Nature Medicine 26, 1070–1076(2020)](https://www.nature.com/articles/s41591-020-0944-y) | Yes |  |
| 5 | Chua et al. | EGAS00001004481 | NB | [Nature Biotechnology](https://www.nature.com/articles/s41587-020-0602-4) | Yes |  |
| 6 | Wen et al. | PRJCA002413 | PBMC | [Cell Discovery 6, 31 (2020)](https://www.nature.com/articles/s41421-020-0168-9) | Yes |  |
| 7 | Yu et al. | PRJCA002579 | PBMC | [researchsquare](https://www.researchsquare.com/article/rs-25869/v1) | Yes |  |
| 8 | Jiang et al. | NA | PBMC | unpublished | Yes |  |
| 9 | Zhang et al. | PRJCA002564 | PBMC | [Nature Immunology](https://pubmed.ncbi.nlm.nih.gov/32788748/) | Yes |  |
| 10 | Wyler et al. | GSE148729 | Cell Line | [biorxiv](https://www.biorxiv.org/content/10.1101/2020.05.05.079194v1) | No | Cell-line data |
| 11 | Cao et al. | EGAS00001004412 | PBMC | [Cell 182, 1, 73-84.e16](https://www.sciencedirect.com/science/article/pii/S0092867420306206) | No | Couldn’t get access |
| 12 | Zhang et al. | HRA000150/PRJCA002564 | PBMC | NA | No | Couldn’t get access |
| 13 | Xue et al. | HRA000216 | PBMC | NA | No | Couldn’t get access |
| 14 | Kun et al. | HRA000172 | PBMC | [biorxiv](https://www.biorxiv.org/content/10.1101/2020.04.08.029769v2) | No | Couldn’t get access |
| 15 | Xue et al. | HRA000225 | PBMC | NA | No | Couldn’t get access |
| 16 | Zhu et al. | CNP0001102 | PBMC | [Immunity](https://www.cell.com/immunity/fulltext/S1074-7613(20)30316-2) | No | Couldn’t get access |

**Supplementary Table 2. Simplification of the stage definition**

| Dataset | Stage annotation in original study | Stage annotation in current study | Any additional comment |
| --- | --- | --- | --- |
| 10X | Healthy | Healthy |  |
| Lee et al.[^1^](https://paperpile.com/c/fq4a9u/SInZk) | Asymptomatic | Asymptomatic |  |
|  | Healthy | Healthy |  |
|  | Influenza | Influenza |  |
|  | Mild | Mild | This is kept as Mild, as it shows a different distribution from other moderate samples. |
|  | Severe | Severe |  |
| Wilk et al.[^2^](https://paperpile.com/c/fq4a9u/0wrEn) | Healthy | Healthy |  |
|  | NonVent | Moderate |  |
|  | Vent | Severe |  |
| Zhang et al.[^3^](https://paperpile.com/c/fq4a9u/3KfYA) | Healthy | Healthy |  |
|  | Moderate | Moderate |  |
|  | Severe | Severe |  |
|  | convalescent | convalescent |  |
| Wen et al.[^4^](https://paperpile.com/c/fq4a9u/fC7uS) | Healthy | Healthy |  |
|  | early recovery | convalescent |  |
|  | late recovery | late recovery |  |
| Yu et al.[^5^](https://paperpile.com/c/fq4a9u/mXGPk) | Healthy | Healthy |  |
|  | Convalescence Mild | convalescent |  |
|  | P&C Mild | Post Mild |  |
|  | Post Mild | Post Mild |  |
| Jiang et al.[^6^](https://paperpile.com/c/fq4a9u/wCvws) | early recovery | convalescent |  |
| Liao et al.[^7^](https://paperpile.com/c/fq4a9u/iNtel) | Healthy | Healthy |  |
|  | Moderate | Moderate |  |
|  | Severe | Severe |  |
| He et al.[^8^](https://paperpile.com/c/fq4a9u/CjM2F) | Severe | Severe |  |
| Chua et al.[^9^](https://paperpile.com/c/fq4a9u/btvog) | Healthy | Healthy |  |
|  | Moderate | Moderate |  |
|  | critical | Severe |  |

**Supplementary Table 3. The self-projection accuracy of the 5 cell populations.**

| **Self-projection accuracy** | **Cross_validation** | **Training set** | **Test se** |
| --- | --- | --- | --- |
| **B** | 0.9535 | 0.9608 | 0.9200 |
| **Lymphoid** | 0.9509 | 0.9671 | 0.9492 |
| **Myeloid** | 0.9567 | 0.9688 | 0.9404 |
| **Epithelial** | 0.9873 | 0.9922 | 0.9863 |
| **Platelets** | 0.9934 | 0.9970 | 0.9957 |

**Supplementary Table 4. Extended version of Table 2 with potential reasons for non-reproducibility.** TCR: T-cell receptor; TNF: tumor necrosis factor; IL: interleukin; HLA: human leukocyte antigen; CD8eff T-cell: CD8^+^ effector T-cell; Supp: Supplementary. Please note that the conclusions by Ren et al. 2021[^10^](https://paperpile.com/c/fq4a9u/mqnAe) have also been included in this table.

| Study | Key finding(s) in COVID-19 patients compared to healthy controls | Reproduced in the original dataset | Consistently reproduced in all other dataset(s) considered for comparison | Potential reason(s) in case of non-reproducibility or additional comments |
| --- | --- | --- | --- | --- |
| Wen et al. [^4^](https://paperpile.com/c/fq4a9u/fC7uS) | Decreased CD4^+^ and CD8^+^ T-cells (data not shown) | Yes | Yes | - Possibility of CD4^+^ T cell death in severe stage - Possibility of T-cell migration from PBMC of COVID-19 patients to infected tissue such as BALF of the patient (may explain the increase in case of Liao dataset) |
|  | Decreased T-cell clonal expansion in convalescent patients compared to healthy controls (Supp Fig. 15c) | No | No | - The healthy controls from Zhang et al. 2020[^3^](https://paperpile.com/c/fq4a9u/3KfYA) seemed to be different from the healthy controls from Wen et al. 2020[^4^](https://paperpile.com/c/fq4a9u/fC7uS) in terms of percentage of clonally expanded T-cells, thereby making it difficult to find a decrease in convalescent patients compared to healthy controls as reported by Wen et al. 2020[^4^](https://paperpile.com/c/fq4a9u/fC7uS). - Patient specific variability in fighting COVID-19 infection |
|  | Increased CD14^+^ monocytes (Supp Fig. 9h) | Yes | No | - Patient-specific variability in fighting COVID-19 infection - Lack of comparable stages in other datasets |
|  | Increased B-cell clonal expansion (Supp Fig. 17b) | Yes | Yes |  |
|  | Increased Plasma cells (Fig. 3f, Supp Fig. 9h) | Yes | Yes | - Only a small number of Plasma cells have been detected in the data from healthy donors. |
|  | Decreased naïve B-cells (Supp Fig. 9c, 9d) | No | No | - An increase is observed instead |
| Zhang et al. [^3^](https://paperpile.com/c/fq4a9u/3KfYA) | IFN-ɑ response upregulation (Supp Figs. 10d, 10e and 11) | Yes | Yes |  |
|  | Increased T-cell clonal expansion (Supp Fig. 15c) | Yes | No | - Lack of another PBMC TCR dataset with samples from severe and moderate patients. - Although the moderate and severe patients of Zhang et al. 2020[^3^](https://paperpile.com/c/fq4a9u/3KfYA) and Liao et al. 2020[^7^](https://paperpile.com/c/fq4a9u/iNtel) seemed to share a similar percentage range of clonally expanded T-cells, Liao et al. 2020[^7^](https://paperpile.com/c/fq4a9u/iNtel) lacked the TCR data corresponding to healthy controls from BALF tissue, making it difficult to draw reliable conclusions. - The differences between individuals were found to be large, and in some datasets greater than the differences between groups (Supp. Fig. 15c). This seems to indicate that there are multiple factors that may affect TCR clonal expansion. |
|  | Increased CD8eff T-cell clonal expansion (Supp Figs. 15a, 15b and 16b) | Yes | Yes | In per study analysis, only 2 samples in Zhang dataset and 2 samples in Liao dataset had TCR data on more than 100 CD8^+^ effector T-cells (Supp Fig S16a). As all these samples were derived from COVID-19 patients, we marked it as consistent. Please note that these two datasets are derived from two different tissues. |
|  | Increased Plasma cells (Fig. 3f) | Yes | Yes |  |
|  | Decreased memory B-cells (Supp Fig. 9b) | Yes | No | Patient-specific variability in fighting COVID-19 infection |
| Lee et al. [^1^](https://paperpile.com/c/fq4a9u/SInZk) | TNF/IL-1β driven inflammatory response upregulation (data not shown) | No | No | - Not all the cell types are consistent. - In the Lee dataset, TNF and IL1B show higher expression in moderate samples but not too high in severe samples. - In case of other datasets, different studies are different. e.g., Wilk T cells do not express significant TNF/IL1B. |
|  | Co-existence of type I IFN response with TNF/IL-1β–driven inflammation in severe COVID-19 patients (data not shown) | Yes, but not sharp contrast with milder | No | Patient-specific variability in fighting COVID-19 infection |
| Wilk et al. [^2^](https://paperpile.com/c/fq4a9u/0wrEn) | Developing neutrophil population from plasmablasts in severe COVID-19 patients (Supp Figs. 13, 14) | Yes | No | - Developing neutrophil markers were present in the cell subpopulation annotated as “Epithelial (RACK1)” in current study - “Epithelial (RACK1)” represented a broad set of cells including red blood cells - Low number of Plasma and “Epithelial (RACK1)” cells captured by other datasets compared to Wilk dataset (n=2862 cells) - Seq-Well platform for scRNA-seq used in Wilk dataset compared to 10X used in other studies - The datasets Liao et al. 2020[^7^](https://paperpile.com/c/fq4a9u/iNtel) and Chua et al. 2020[^9^](https://paperpile.com/c/fq4a9u/btvog) were derived from BALF and nasopharyngeal/bronchial tissues, respectively, compared to others derived from PBMC. - Might correspond to patient-specific variability in fighting COVID-19 infection. |
|  | HLA-class II downregulation in CD14^+^ monocytes (Supp Fig. 10f) | Yes | Yes | Significant downregulation in severe COVID-19 patients compared to healthy controls also observed in CD16^+^ monocytes dendritic cells (DCs) and plasmacytoid dendritic cells (pDCs) but not in NK cells, naive B-cells or memory B-cells (Supp Fig S12) |
|  | Heterogenous ISG module upregulation in CD14^+^ monocytes (Supp Fig. 10g) | Yes | No | - Reproducible in Wilk, Zhang and Liao datasets. - Downregulation in Lee dataset instead. - No statistically significant difference in Chua dataset. - Might correspond to patient-specific variability in fighting COVID-19 infection. |
|  | Presence of type I IFN driven inflammatory signatures in CD14^+^ monocytes (Supp Fig. 10d, 10e) | Yes | Yes |  |
|  | Lack of substantial expression of pro-inflammatory cytokine genes (*TNF, IL6, IL1B, CCL3, CCL4 or CXCL2*) in CD14^+^ monocytes (data not shown) | Yes | No | CD14+ monocytes(FOSB) are not found in Wilk, but found in others (Zhang Lee, Chua). These cells express: TNF, IL1B, CCL3, CCL4, CXCL2 |
| Liao et al. [^7^](https://paperpile.com/c/fq4a9u/iNtel) | Increased CD8^+^ T-cell clonal expansion in moderate COVID-19 patients compared to severe (Supp Fig. 15d and 16b) | No | No | - Additional cut-off of 100 cells per cell subpopulation per sample was applied which led to loss of samples in the Liao dataset (Supp Fig. 16a) - Only Zhang and Liao dataset had TCR data from moderate and severe stages but they are both from different tissues; PBMC and BALF respectively. - No significant difference in CD8^+^ T-cell clonal expansion observed between moderate and severe stages - Patient-specific variability in fighting COVID-19 infection |
| Chua et al. [^9^](https://paperpile.com/c/fq4a9u/btvog) | Activated macrophages expressing inflammatory chemokines including *CCL2, CCL3, CCL20, CXCL1, CXCL3, CXCL10, IL8, IL1B* and *TNF* in severe patients (data not shown) | Yes | No | - Not CCL2 but CCL3, CXCL10 are correct. - Others are weak. - Different studies captured different populations. |
| Ren et al. [^10^](https://paperpile.com/c/fq4a9u/mqnAe) | Increased CD14^+^ monocytes in severe COVID-19 patients (Supp Fig. 9g) | - | No | - Not elevated in Zhang (PBMC) and He (BALF) dataset - Patient specific variability - Lack of healthy samples from BALF - Limited sample size |
|  | Increased Megakaryocytes in severe COVID-19 patients (Supp Fig. 9i) | - | No | - Not elevated in Zhang (PBMC) and He (BALF) dataset - Patient specific variability - Lack of healthy samples from BALF - Limited sample size |
|  | No significant change in NK cells (Supp Fig. 9j) | - | Yes |  |
|  | Increased B-cells in severe COVID-19 patients (Supp Fig. 9a) | - | No | - Patient specific variability - Limited sample size |
|  | Decreased γδ T-cells (Supp Fig. 9k) | - | No | - No decrease in moderate patients of Zhang (PBMC), Liao (BALF) and Chua (NB) datasets - Patient specific variability - Limited sample size |
|  | Increased Proliferating T-cells (Supp Fig. 9l) | - | Yes |  |
|  | Decreased DCs (Supp Fig. 9e) | - | No | - Decreased in 4/6 datasets tested but not in Wilk (PBMC) and He (BALF) datasets. - Lack of healthy samples in He (BALF) dataset - Patient specific variability - Limited sample size |
|  | Cytokine storms mediated by CD14^+^ monocytes (Supp Fig. 10) | - | Yes | - Pathways related to cytokine storm identified in Gene Ontology (GO) analysis of common differentially expressed genes in CD14^+^ monocytes |
|  | Association of naive CD8^+^ T-cells with age (Supp Fig. 19a) | - | No | - Heterogeneity in sample collection, for example, most patients from the Lee dataset are above the age of 50 years. - 12 out of 15 patients from Lee et al. 2020[^1^](https://paperpile.com/c/fq4a9u/SInZk) were mostly in the age group of 50-75 years and 18 out of 22 patients from Zhang et al. 2020[^3^](https://paperpile.com/c/fq4a9u/3KfYA) were mostly in the age group of 29-60 years leaving us with only 2 samples on either extremes to properly deduce the slope of a fitted line. - Lack of age information from Wen[^4^](https://paperpile.com/c/fq4a9u/fC7uS) and Wilk[^2^](https://paperpile.com/c/fq4a9u/0wrEn) datasets as given in Supp data 1 - Limited sample size in each dataset considered for meta-analysis - Difference in data analysis (for example, no correction for technical covariates is performed in this meta-analysis due to absence of relevant information) |
|  | Presence of sex associated T-cell subsets (Supp Fig. 19b) | - | No | - Difference in cell-type annotations to perform direct comparison - Heterogeneity in sample collection, for example, Wilk dataset has samples from only 2 females (both healthy) - Limited sample size in each dataset considered for meta-analysis |

# Supplementary Figures


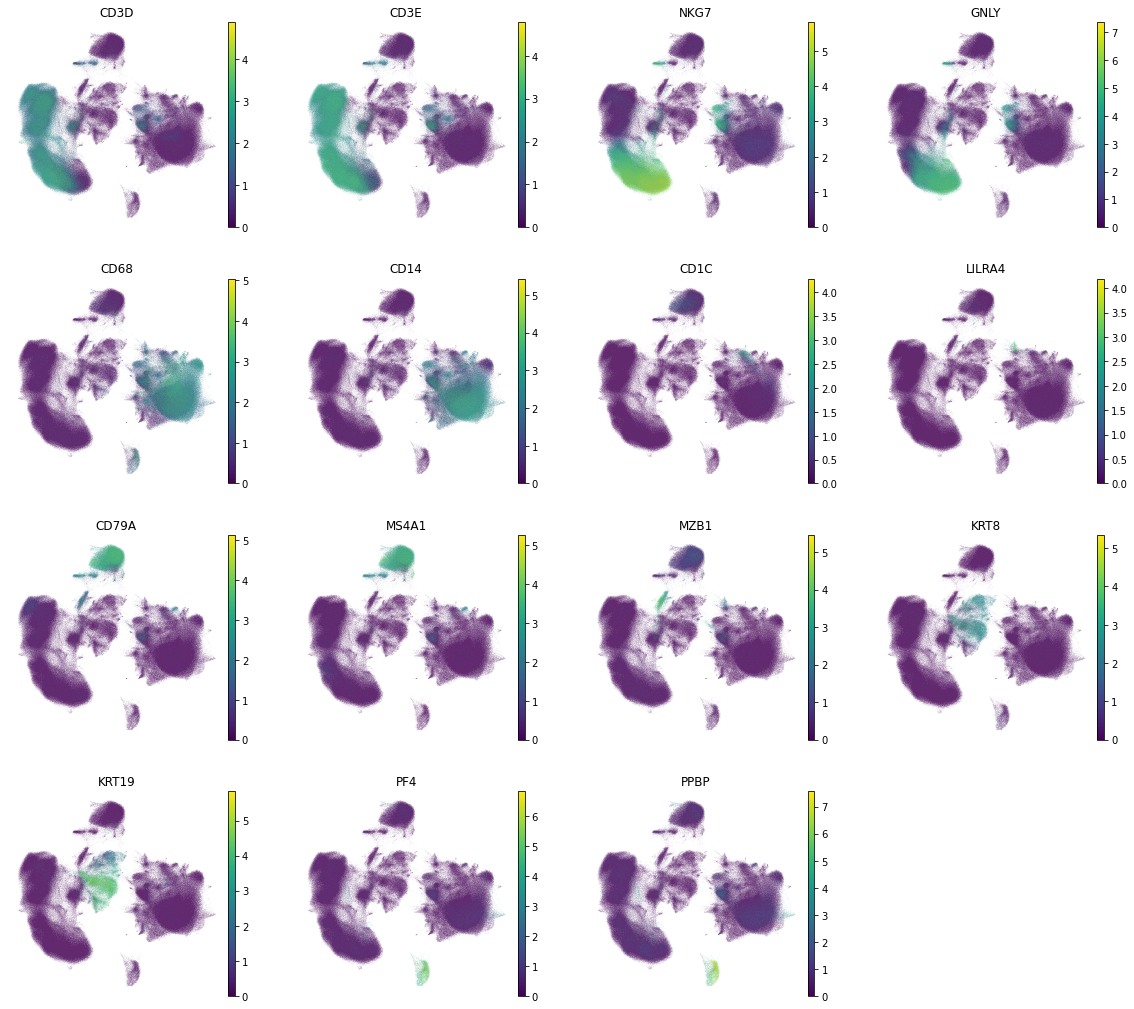


**Supplementary Figure 1. Marker gene expression for the 5 cell populations.** The UMAPs show the marker gene expression of Lymphoid cells (*CD3D, CD3E, NKG7, GNLY*), Myeloid cells(*CD68, CD14, CD1C, LILRA4*), B cells (*CD79A, MS4A1, MZB1*), Epithelial cells (*KRT8, KRT19*) and Platelets (*PF4, PPBP*).


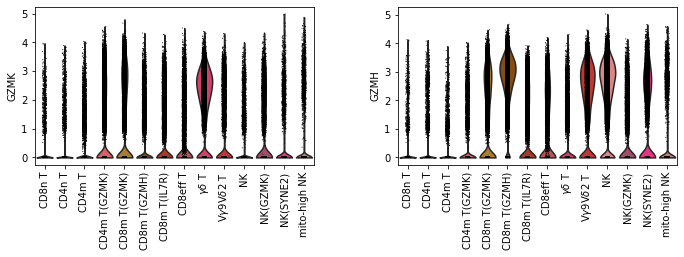


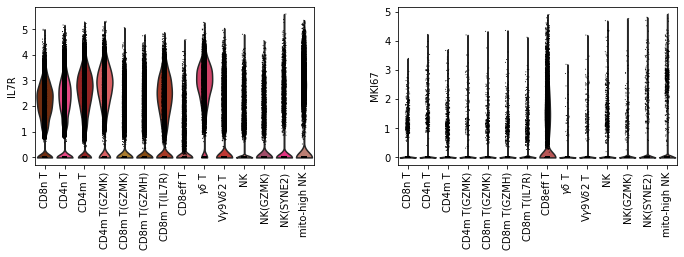


**Supplementary Figure 2.** **The violin plots of the marker gene expressions for CD8 T cell subpopulations.** *GZMK, GZMH, IL7R* and *MKI67* are used to distinguish CD8m T(GZMK), CD8m T(GZMH), CD8m T(IL7R) and CD8eff T cells.


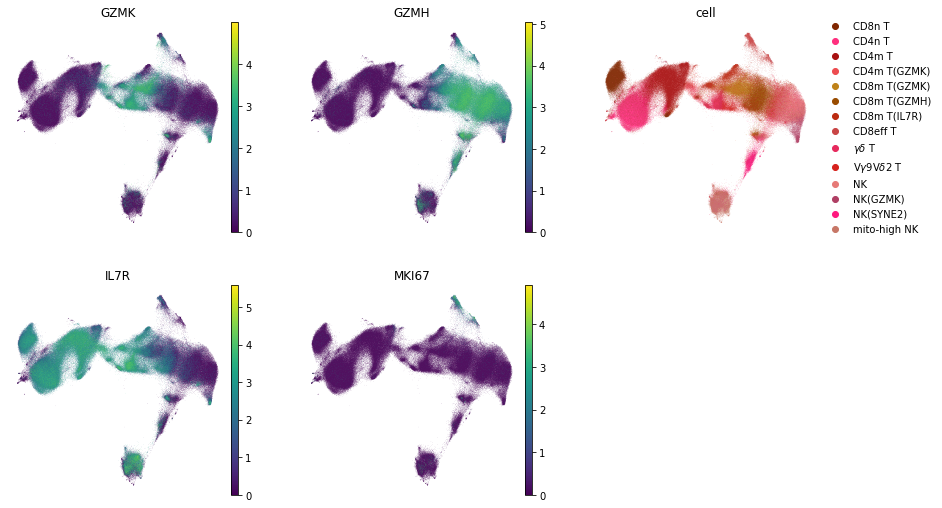


**Supplementary Figure 3. The UMAP plots of the marker gene expressions for CD8 T cell populations.** *GZMK, GZMH, IL7R* and *MKI67* are used to distinguish CD8m T(GZMK), CD8m T(GZMH), CD8m T(IL7R) and CD8eff T cells. *GZMH* and *GZMK* not only show good discrimination of the CD8 T cell subpopulations, but also discriminates the NK cell populations.


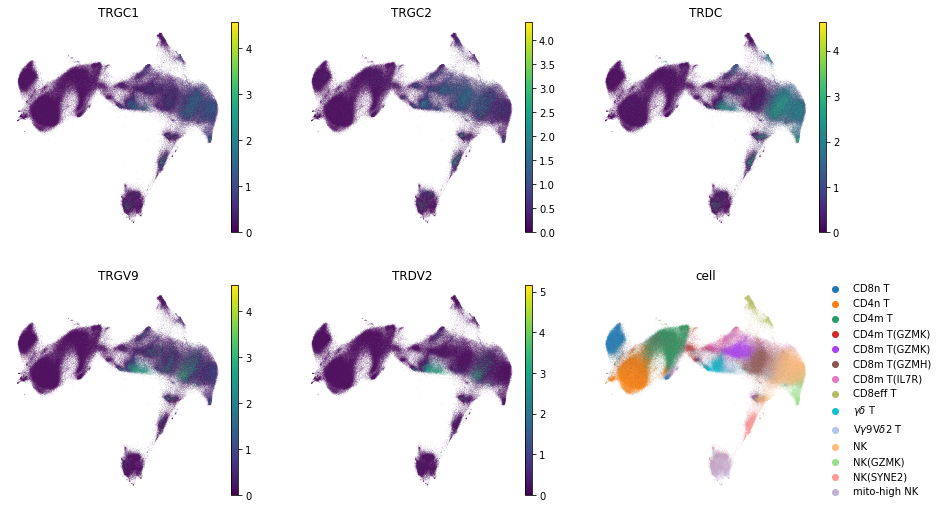
**Supplementary Figure 4. The UMAP plots of the marker gene expressions for γδ T cell populations.** *TRGC1, TRGC2* and *TRDC* are the marker genes suggested by Wilk et al.[^2^](https://paperpile.com/c/fq4a9u/0wrEn), while *TRGV9* and *TRDV2* are suggested by Zhang et al.[^3^](https://paperpile.com/c/fq4a9u/3KfYA)

##
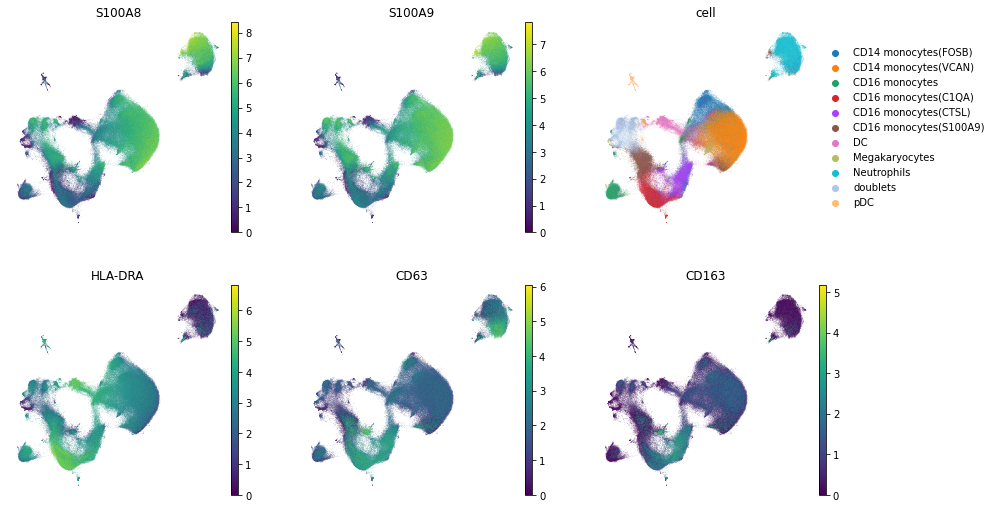


**Supplementary Figure 5. The UMAP plots of the marker gene expressions for CD16 monocytes populations.** The CD16^+^ monocytes express high *FCGR3A* (*CD16*), while CD16 monocytes(C1QA) not only express the complement proteins (*C1QA, C1QB, C1QC*) but also *HLA-DRA* and *CD63*. The CD16 monocytes(CTSL) cluster expresses *CTSL* and *CD163*, the CD16 monocytes(S100A9) cluster expresses *S100A8* and *S100A9.*


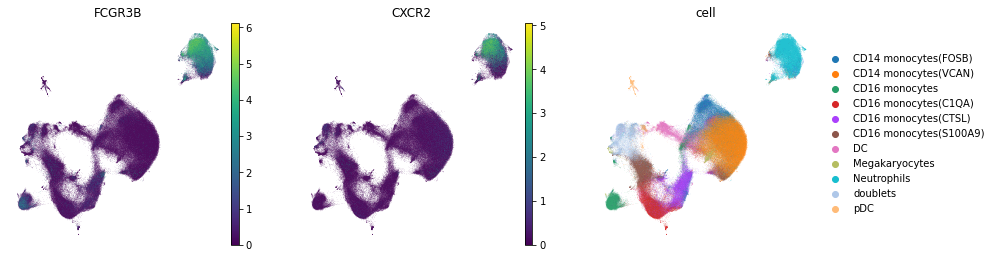


**Supplementary Figure 6. The UMAP plots of the marker gene expressions for neutrophils.**

**
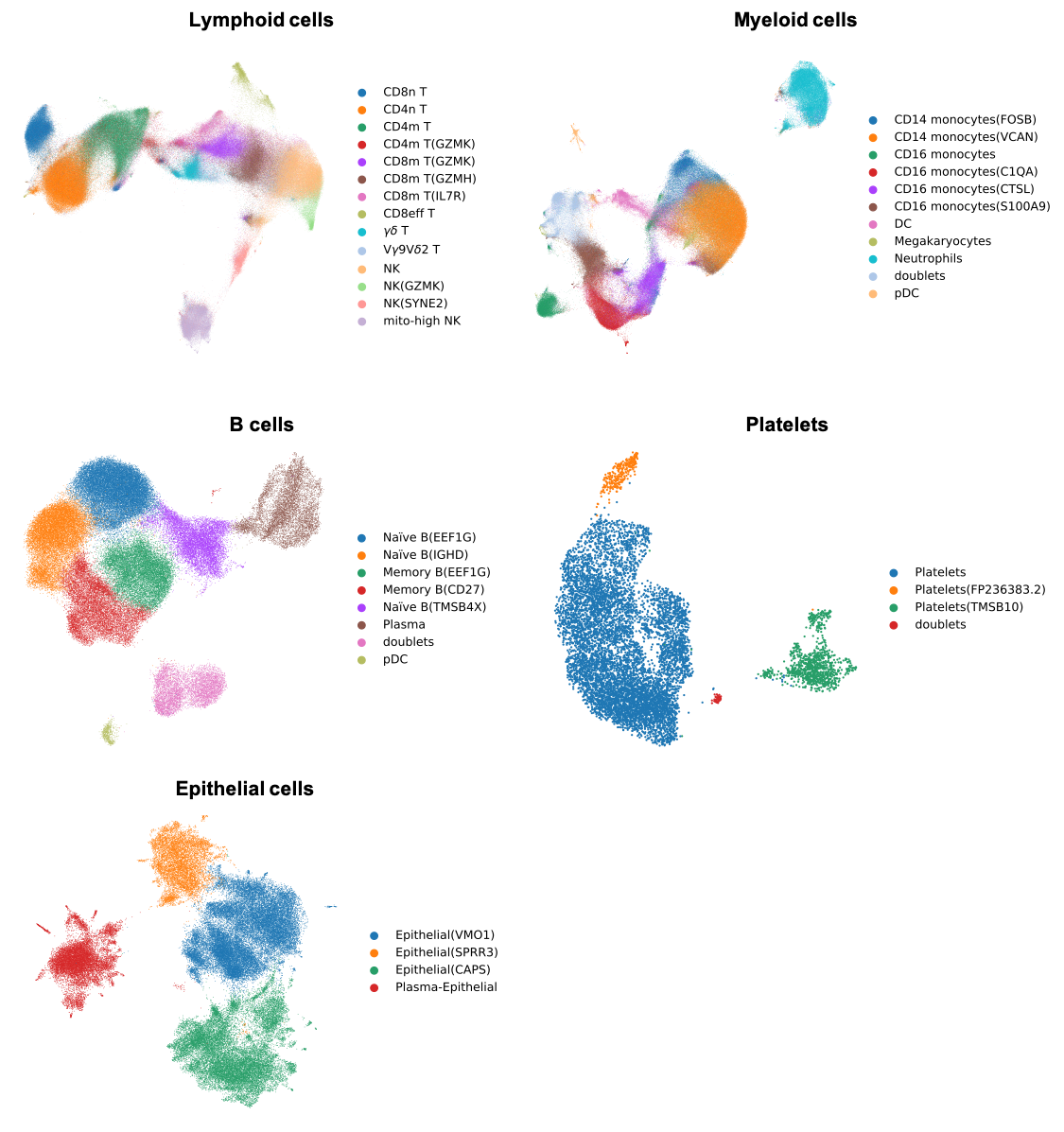
**

**Supplementary Figure 7. UMAP visualisation of the cell subpopulations in the five main cell types.**

**
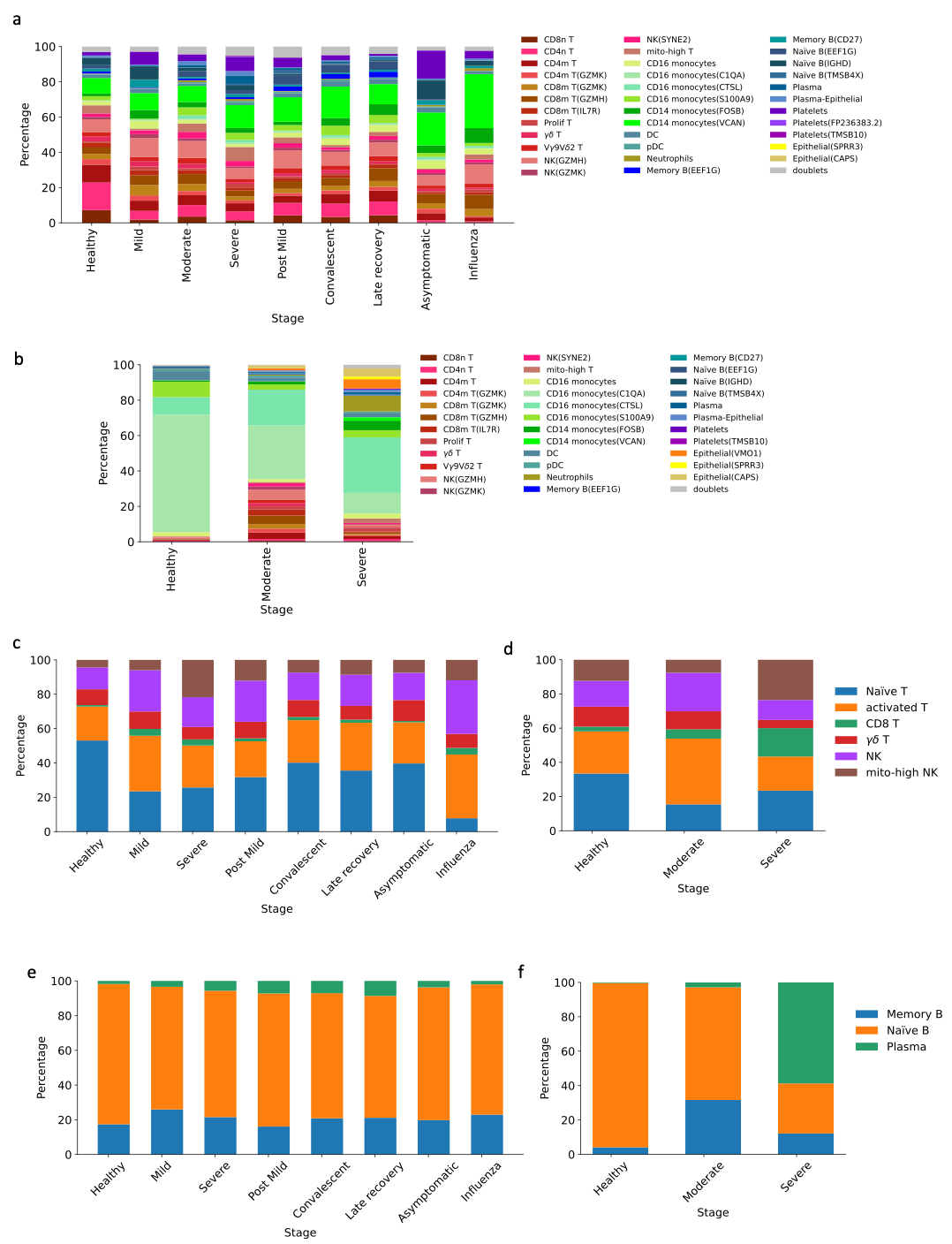
**

**Supplementary Figure 8. The cell proportion changes BALF and PBMC. a) and** **b)** Proportion changes of all cells in PBMC and BALF, respectively. **c)** **and** **d)** Lymphoid cell proportion changes in PBMC and BALF, respectively. **e)** **and** **f)** B-cell proportion changes in PBMC and BALF, respectively.


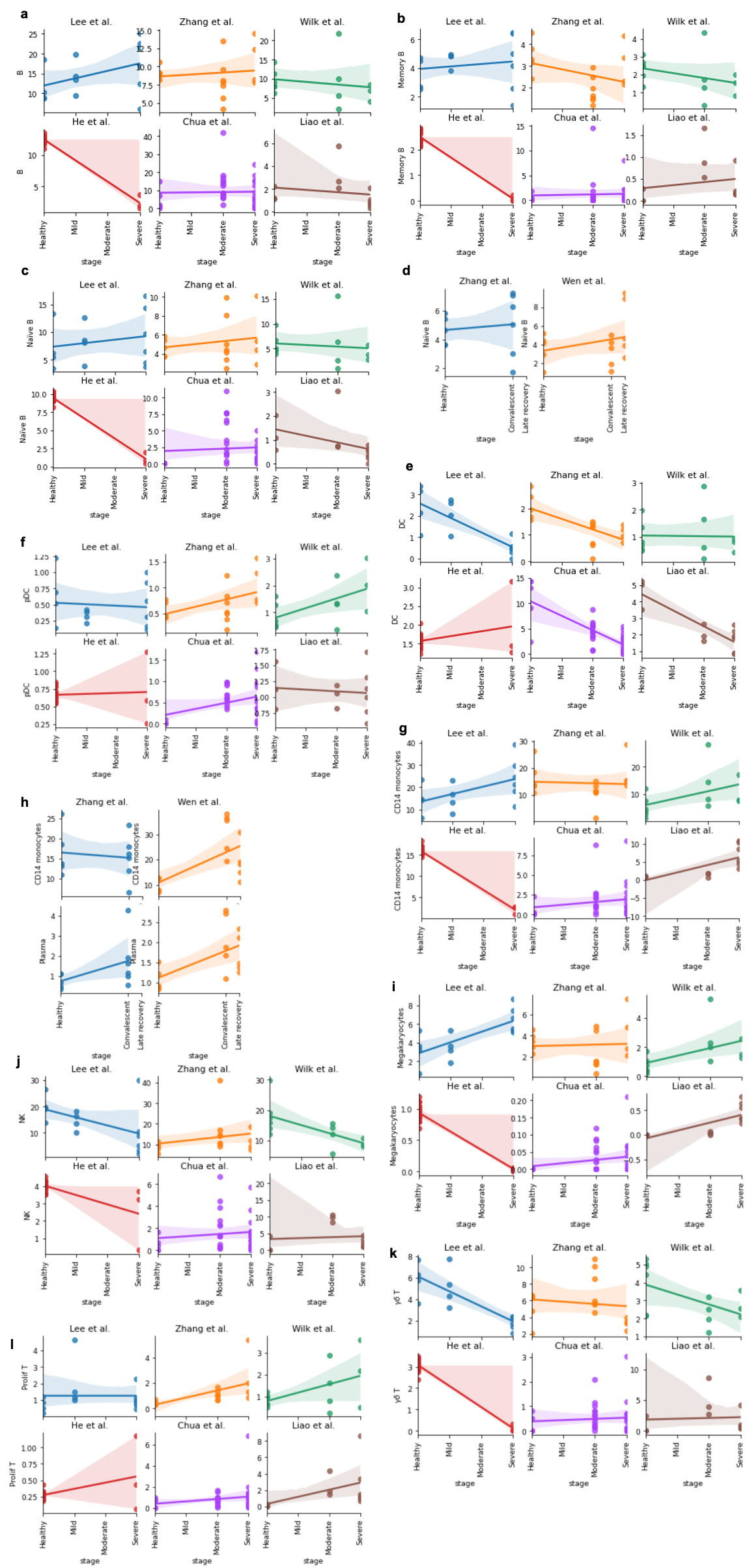


**Supplementary Figure 9. The cell proportion changes across multiple datasets and stages.** In **a)** all B cells; **b)** memory B-cells; **c)** naïve B-cells across datasets with stages healthy, (mild), moderate and severe; **d)** naïve B-cells across datasets with stages healthy, convalescent (and late recovery); **e)** dendritic cells (DC); **f)** Plasmacytoid dendritic cells (pDC); **g)** CD14^+^ monocytes; **h)** plasma cells and CD14^+^ monocytes with stages healthy, convalescent (and late recovery); **i)** megakaryocytes; **j)** NK cells; **k)** γδ T-cells; **l)** Prolif T-cells

##
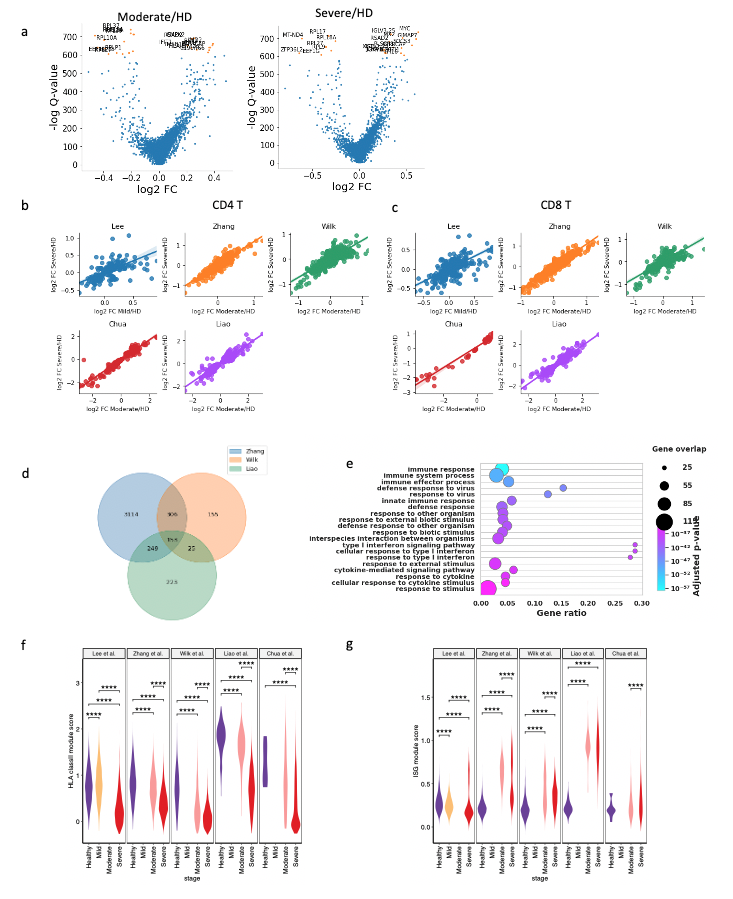


**Supplementary Figure 10. Differentially expressed genes and regulated pathways. a)** The volcano plots show the differential expressions between: 1) moderate patients and healthy donors; 2) severe patients and healthy donors; **b)** For CD4 T cells, the dot plots show the correlation between the log fold change of moderate/healthy and that of severe/healthy in the five studies; **c)** For CD8 T cells, the dot plots show the correlation between the log fold change of moderate/healthy and that of severe/healthy in the five studies; **d)** The venn plot shows the number of upregulated genes in CD14^+^ monocytes overlapped between the three studies. 3822, 639 and 650 genes are upregulated in both moderate samples compared to healthy and severe samples compared to healthy in the Zhang, Wilk and Liao datasets respectively; **e)** The dot plot shows the top upregulated pathways traced from the 153 overlapped genes identified in d). The number of differentially expressed genes (DEGs) that share a particular GO term is represented by the size of the datapoint, the color shows the FDR-corrected p-value, and the x-axis is the ratio of genes in the full DEG set that contain the particular annotation. **f)** Violin plots comparing the HLA class II module score of each CD14^+^ monocytes across studies. All differences were analyzed using two-sided unpaired Wilcoxon rank sum tests with Bonferroni correction and p-values <0.05 are reported. **g)** Violin plots comparing the ISG module score of each CD14^+^ monocytes across studies. All differences were analyzed using two-sided unpaired Wilcoxon rank sum tests with Bonferroni correction and p-values <0.05 are reported.

CD4 T


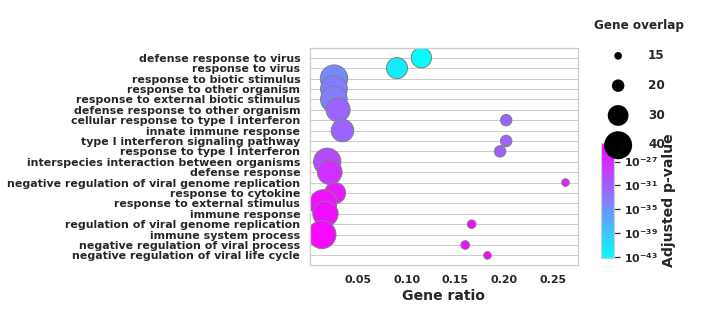


CD8 T


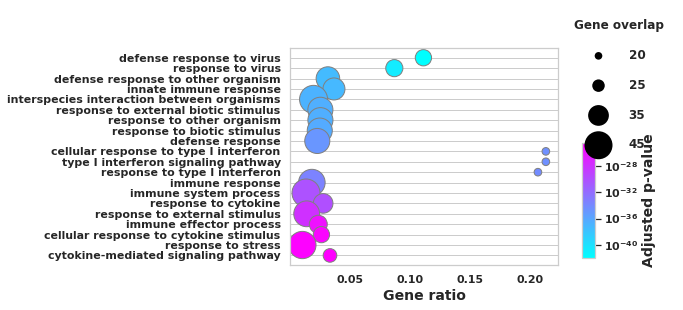


γδ T


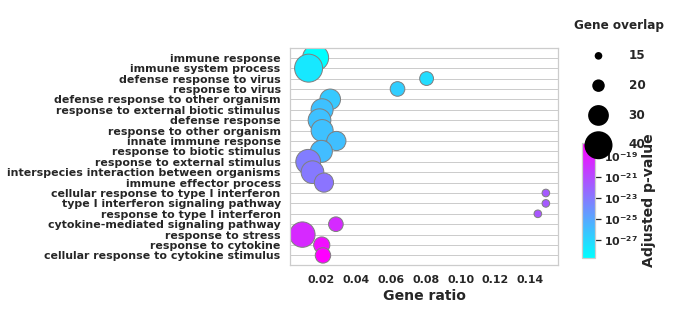


DC


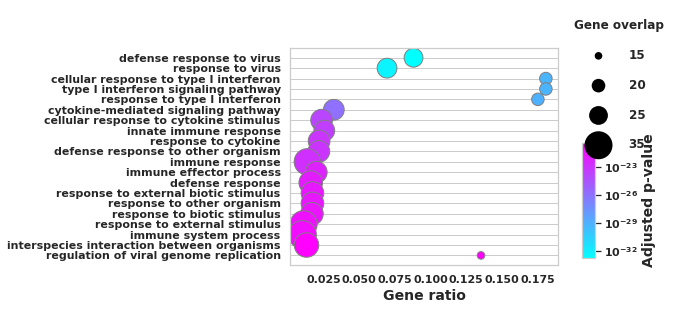


pDC


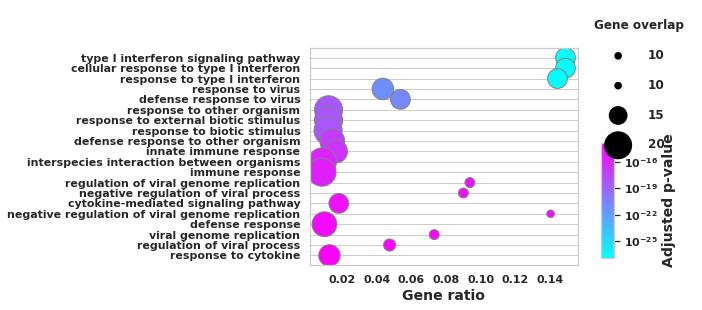


Neutrophils


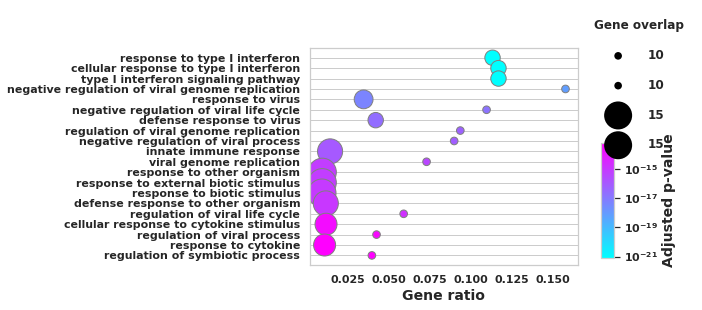


Plasma


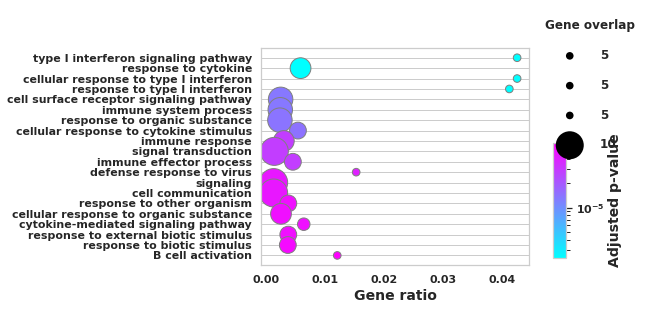


B


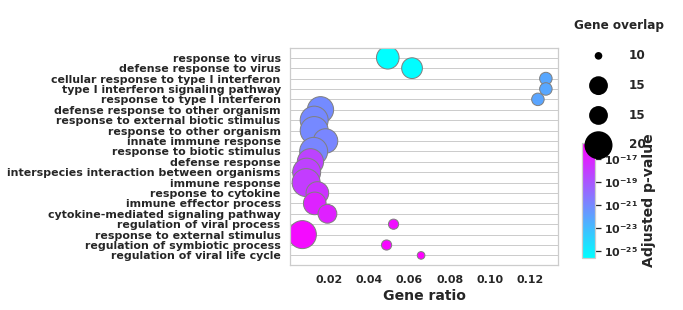


NK


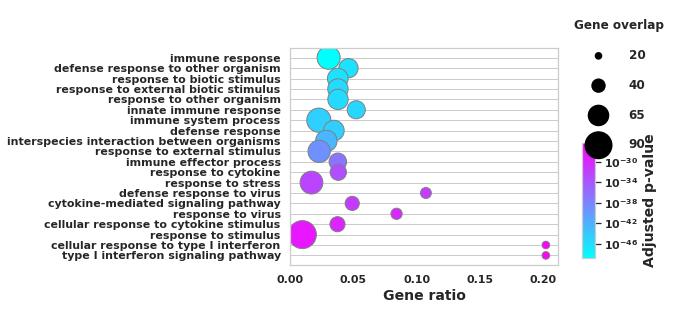


**Supplementary Figure 11. Dot plots showing the top upregulated pathways traced from the overlapping differentially expressed (DE) genes (false discovery rate < 0.01) across multiple datasets in each cell-type.** These DE genes were found to be upregulated in both moderate vs healthy control comparison and severe vs healthy control comparison. They were further identified to be in common when the same analysis was performed in multiple datasets (Zhang, Wilk and Liao datasets)

**
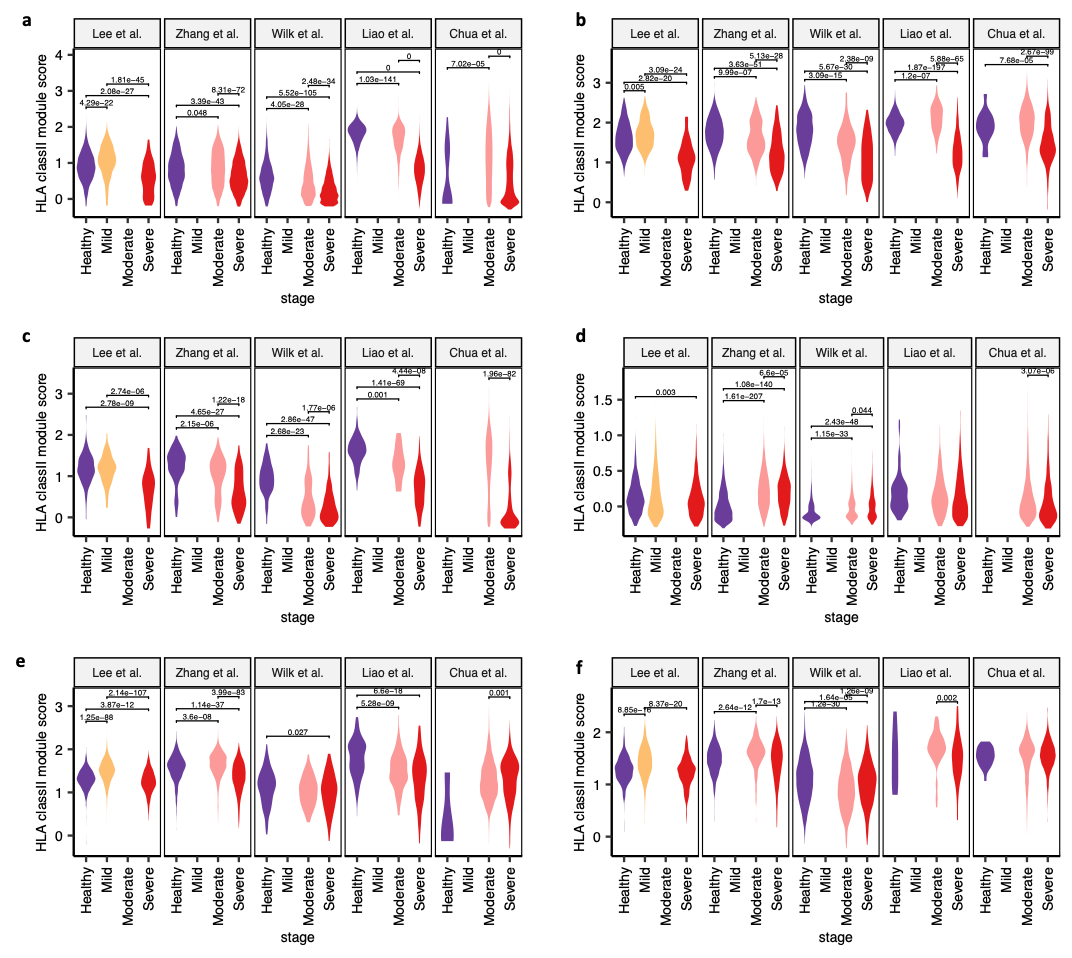
**

**Supplementary Figure 12. Significant HLA class II downregulation in severe COVID-19 patients compared to healthy controls was observed in multiple cell-types across studies. a)** CD16+ monocytes **b)** Dendritic cells **c)** Plasmacytoid dendritic cells **d)** Natural killer cells **e)** Naive B-cells **f)** Memory B-cells. All differences were analyzed using two-sided unpaired Wilcoxon rank sum tests with Bonferroni correction and p-values <0.05 are reported.


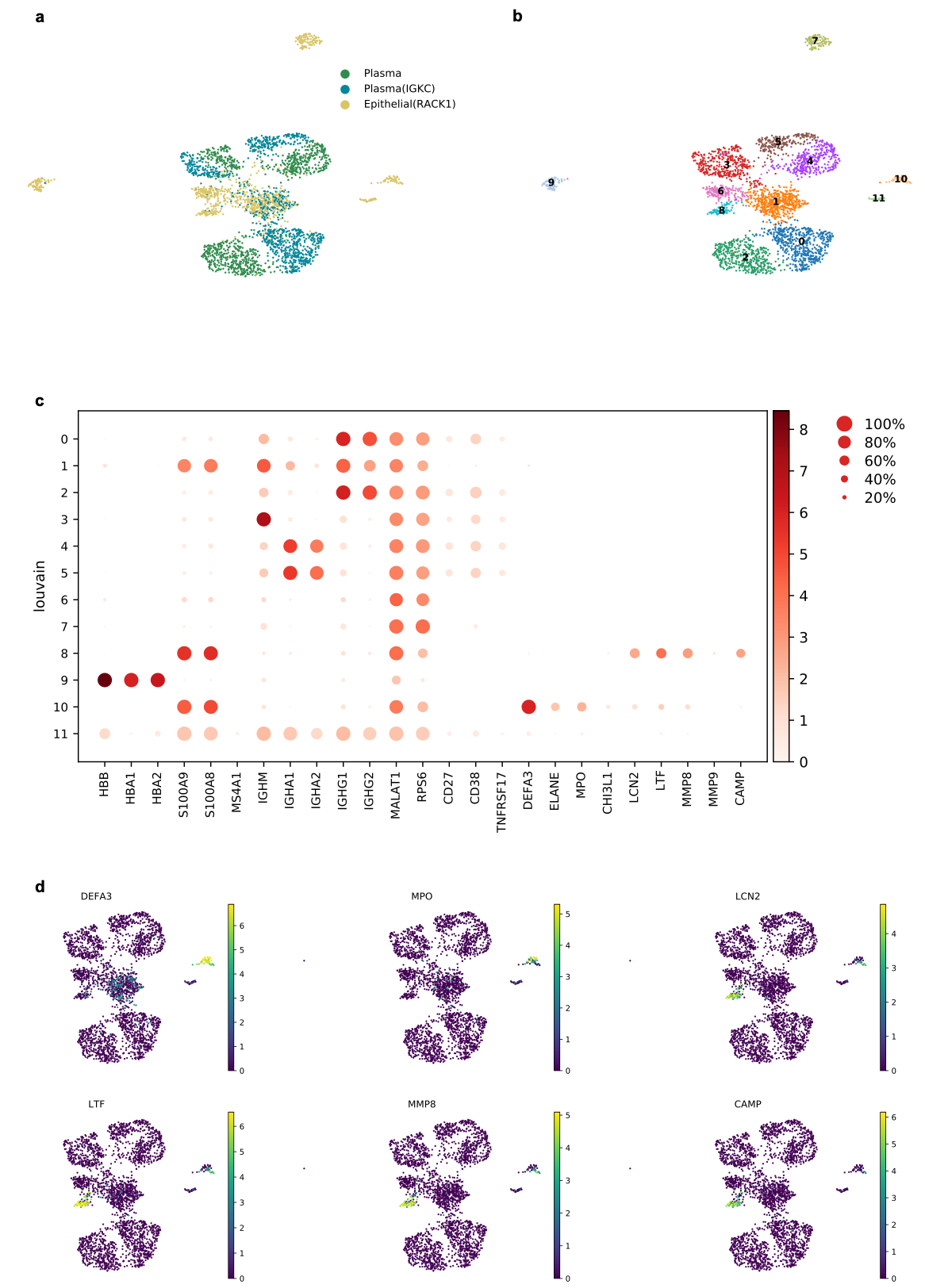


**Supplementary Figure 13. Reproduction of developing neutrophil population in severe COVID-19 patients from Wilk et al. 2020 dataset. a)** UMAP embedding showing the plasma cell and epithelial cell (RACK1) subpopulations in the severe COVID-19 patients of Wilk et al. 2020 dataset (n=2862 cells). **b)** UMAP embedding showing the results of louvain clustering on panel a). **c)** Dotplot showing the marker gene expression in each of the corresponding louvain clusters shown on the y-axis. **d)** UMAP embedding colored by selected developing neutrophil marker genes’ expression where primary granule proteins are encoded by *DEFA3* and *MPO*, secondary granule proteins are encoded by *LCN2* and *LTF* and the tertiary granule proteins are encoded by *MMP8* and *CAMP* as used by Wilk et al. 2020. Note that the louvain cluster 7 and 9 have been removed from this representation to improve the graphics as they don’t represent either plasma cells or developing neutrophil population (panel c).


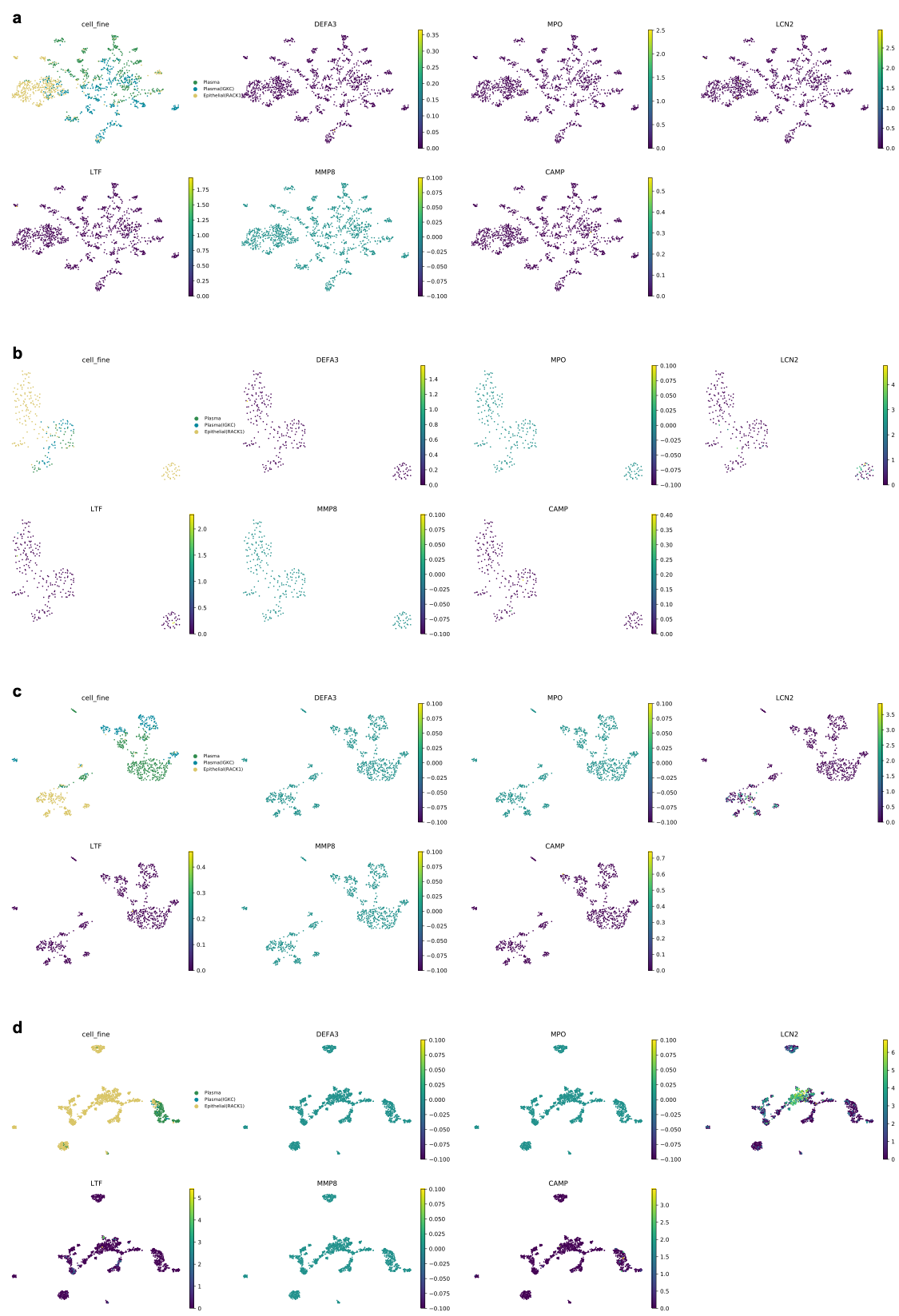


**Supplementary Figure 14. Validation of developing neutrophil population in severe COVID-19 patients from datasets other than Wilk et al. 2020.** UMAP embedding showing the plasma cell and epithelial cell (RACK1) subpopulations in the severe COVID-19 patients of **a)** Zhang et al. 2020 dataset (n=1320 cells) **b)** Lee et al. 2020 dataset (n=245 cells) **c)** Liao et al. 2020 dataset (n=830 cells) **d)** Chua et al. 2020 dataset (n=1884 cells)

##
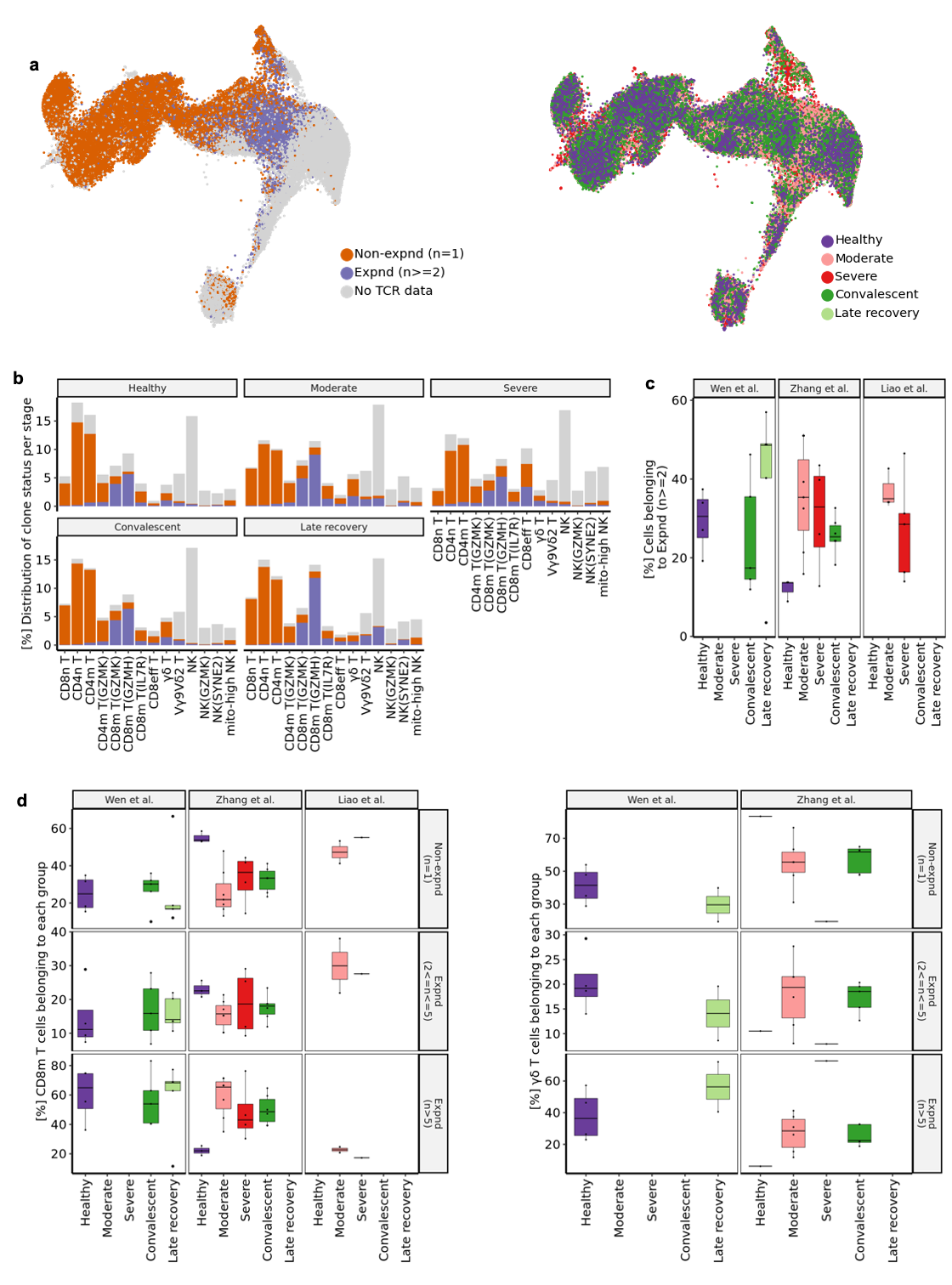


**Supplementary Figure 15. Meta-analysis yields inconclusive results regarding the CD8^+^ T-cell clone expansion in COVID-19 patients compared to healthy.** **a)** UMAP of T cells and NK cells derived from Wen et al. 2020, Zhang et al. 2020 and Liao et al. 2020 (BALF). Colors indicate the clusters of TCR detection and cells belonging to expanded (Expnd) vs non-expanded (Non-expnd) clonotypes (left) and the stage of the patients (right). Here, n denotes the number of clones in each clonotype belonging to that group. **b)** Bar plots showing the percentage of cells at each stage belonging to the specific T-cell subpopulation as well as clone status. Here, grey color denotes no TCR data, orange color denotes non-expanded clone status (Non-expnd) and blue color denotes expanded clone status (Expnd). **c)** Box plots comparing the percentage of all the T-cells belonging to expanded clonotypes (Expnd (n>=2)) at each stage across studies. Colors denote the stage of the patient. All differences were analyzed using two-sided unpaired Wilcoxon rank sum tests with Bonferroni correction and p-values <0.05 are reported. **d)** Box plots comparing the percentage of CD8^+^ memory T-cells (left) and γẟ T-cells (right) belonging to each group of clonotypes at each stage across studies. Here, the three groups of clonotypes are non-expanded clonotypes (Non-expnd (n=1)), expanded clonotypes with greater than 2 and less than 5 clones (Expnd (2<=n<=5)) and expanded clonotypes with greater than 5 clones (Expnd (n>5)). Colors denote the stage of the patient. All differences were analyzed using two-sided unpaired Wilcoxon rank sum tests with Bonferroni correction and p-values <0.05 are reported.

##
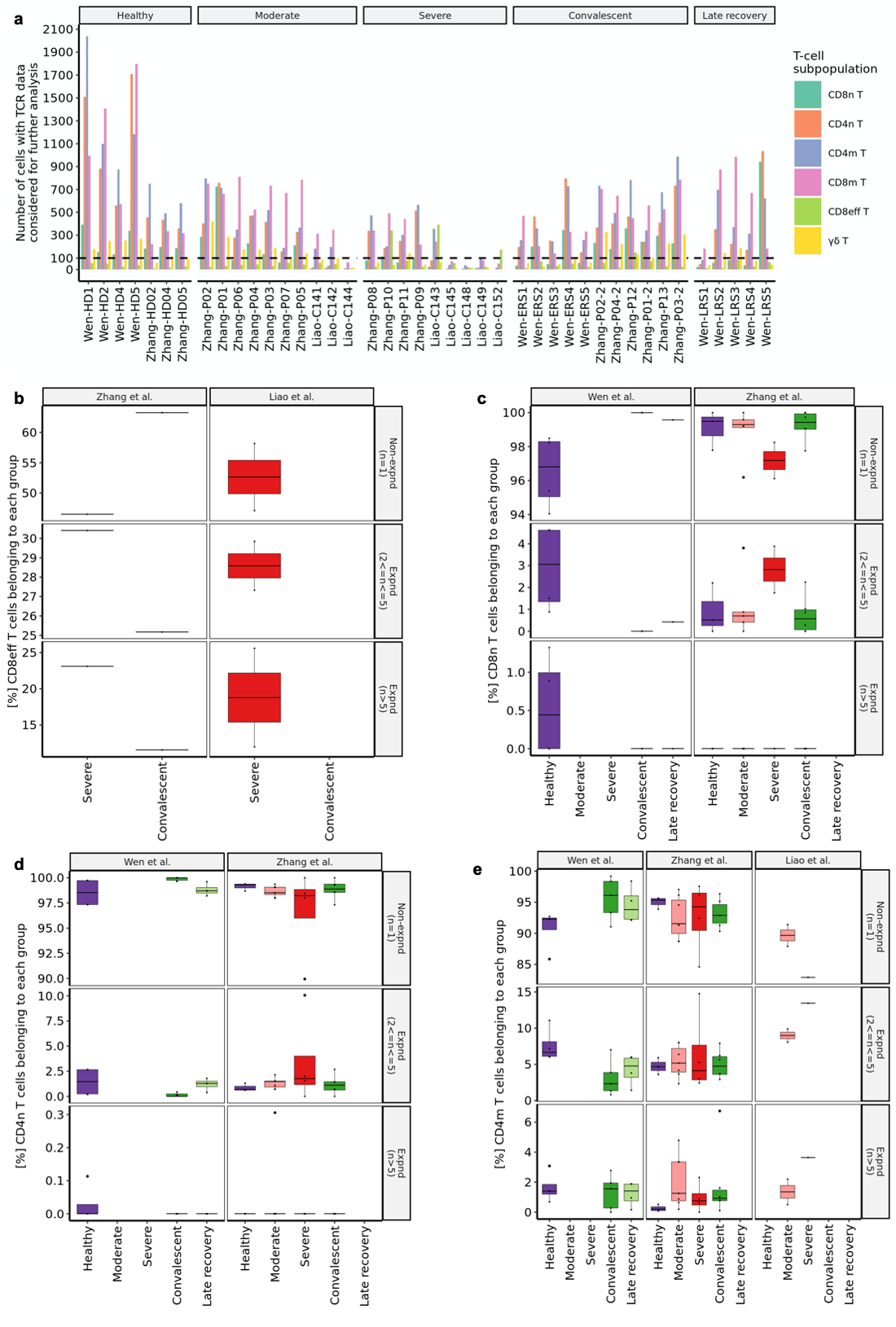


**Supplementary Figure 16. Limitations of current TCR data include insufficient number of effector CD8^+^ T-cells in most of the samples and less than 100 T-cells each in the samples derived from BALF (Liao et al. 2020). a)** Bar plots showing the total number of cells belonging to each T-cell subpopulation in each sample that had the corresponding TCR data, had at least one productive heavy chain and at least one productive light chain. Here, samples with prefix “Wen'' belong to the study Wen et al. 2020, samples with prefix “Zhang” belong to the study Zhang et al. 2020 and samples with prefix “Liao” belong to the study Liao et al. 2020. **b) to** **e)** Box plots comparing the percentage of different T-cells subpopulations belonging to non-expanded clonotypes (Non-expnd (n=1)), expanded clonotypes with greater than 2 and less than 5 clones (Expnd (2<=n<=5)) and expanded clonotypes with greater than 5 clones (Expnd (n>5)) at each stage across four different studies. **b)** Effector CD8^+^ T-cells **c)** naive CD8^+^ T-cells **d)** naive CD4^+^ T-cells **e)** memory CD4^+^ T-cells. Colors denote the stage of the patient. All differences were analyzed using two-sided unpaired Wilcoxon rank sum tests with Bonferroni correction and p-values <0.05 are reported.


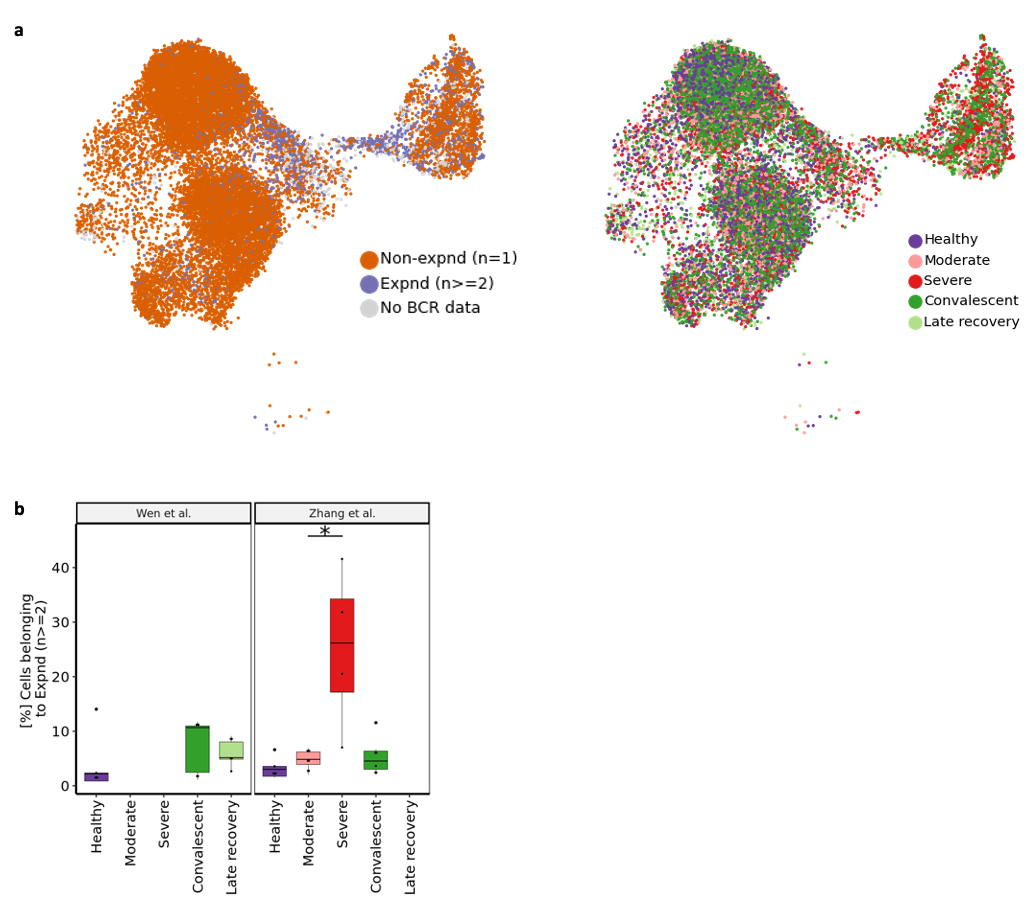


**Supplementary Figure 17. Meta-analysis confirms the presence of expanded B-cell clones in COVID-19 patients. a)** UMAP of B cells derived from Wen et al. 2020 and Zhang et al. 2020. Colors indicate the clusters of BCR detection and cells belonging to expanded (Expnd) vs non-expanded (Non-expnd) clonotypes (left) and the stage of the patients (right). Here, n denotes the number of clones in each clonotype belonging to that group. **b)** Box plots comparing the percentage of all the B-cells belonging to expanded clonotypes (Expnd (n>=2)) at each stage across studies. Colors denote the stage of the patient. All differences were analyzed using two-sided unpaired Wilcoxon rank sum tests with Bonferroni correction and p-values <0.05 are reported.

##
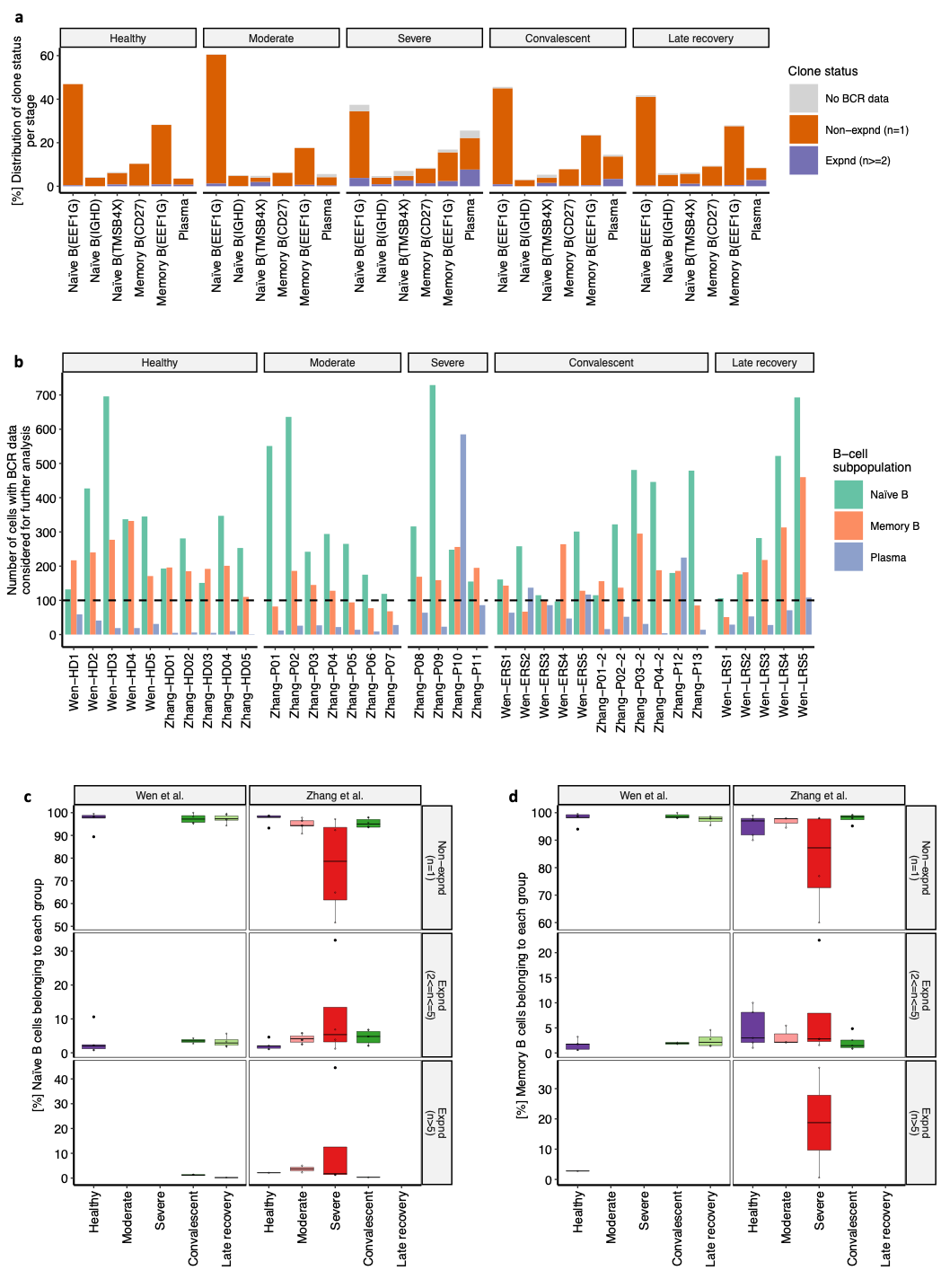


**Supplementary Figure 18. Limitations of current BCR data include insufficient number of Plasma cells and memory B-cells in most of the samples along with presence of study-specific stages. a)** Bar plots showing the percentage of cells at each stage belonging to the specific B-cell subpopulation as well as clone status. Here, grey color denotes no BCR data, orange color denotes non-expanded clone status (Non-expnd) and blue color denotes expanded clone status (Expnd). **b)** Bar plots showing the total number of cells belonging to each B-cell subpopulation in each sample that had the corresponding BCR data, had at least one productive heavy chain and at least one productive light chain. Here, samples with prefix “Wen'' belong to the study Wen et al. 2020 and samples with prefix “Zhang” belong to the study Zhang et al. 2020. **c) to d)** Box plots comparing the percentage of **c)** naïve B-cells and **d)** memory B-cell subpopulations belonging to non-expanded clonotypes (Non-expnd (n=1)), expanded clonotypes with greater than 2 and less than 5 clones (Expnd (2<=n<=5)) and expanded clonotypes with greater than 5 clones (Expnd (n>5)) at each stage across four different studies. Colors denote the stage of the patient. All differences were analyzed using two-sided unpaired Wilcoxon rank sum tests with Bonferroni correction and p-values <0.05 are reported.

##
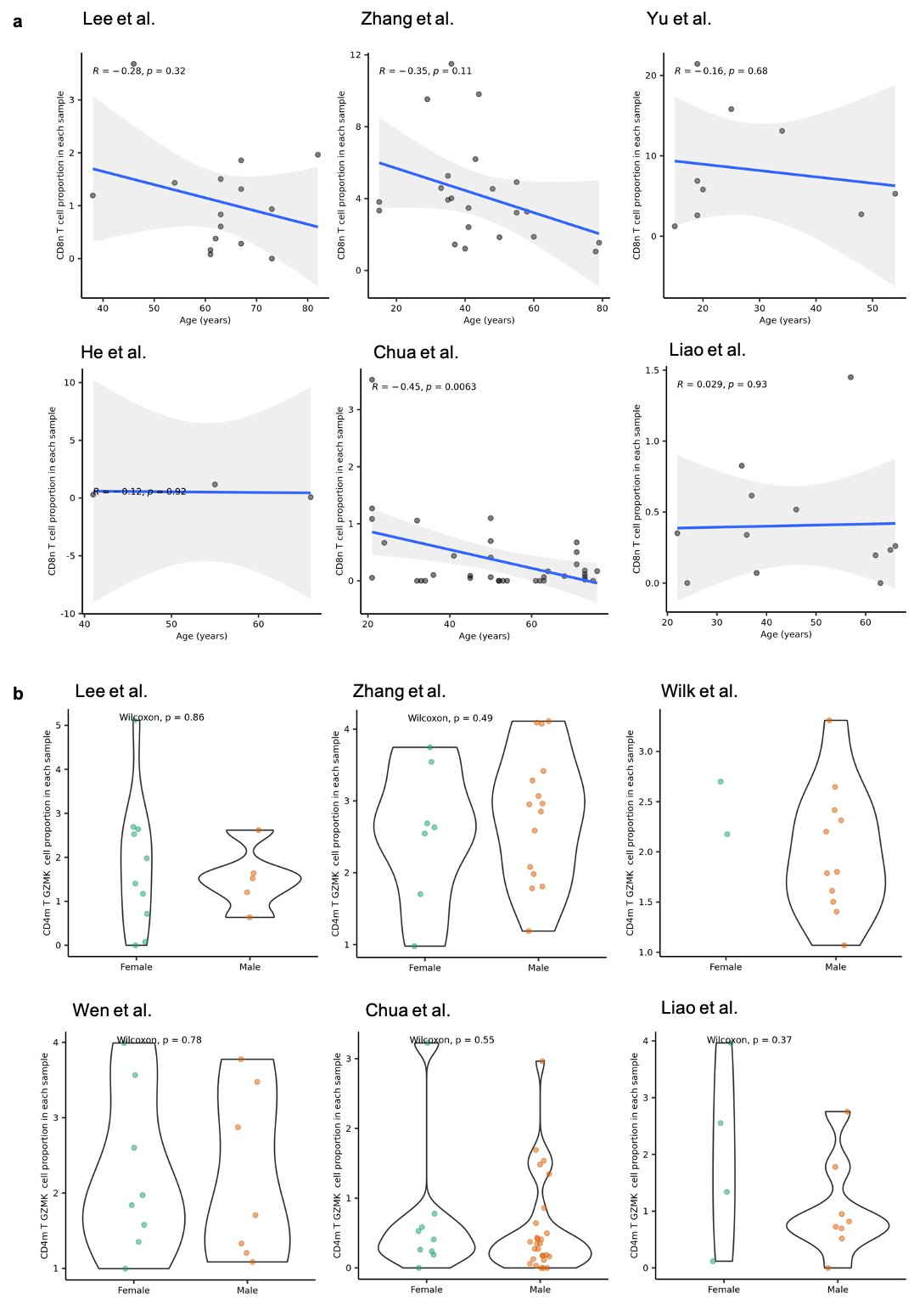


**Supplementary Figure 19. Validation of results from Ren et al. 2021**[^10^](https://paperpile.com/c/fq4a9u/mqnAe)**.** Association of **a)** naive CD8+ T-cells with patient age and **b)** memory CD4+ T-cell (GZMK) with patient sex across datasets.


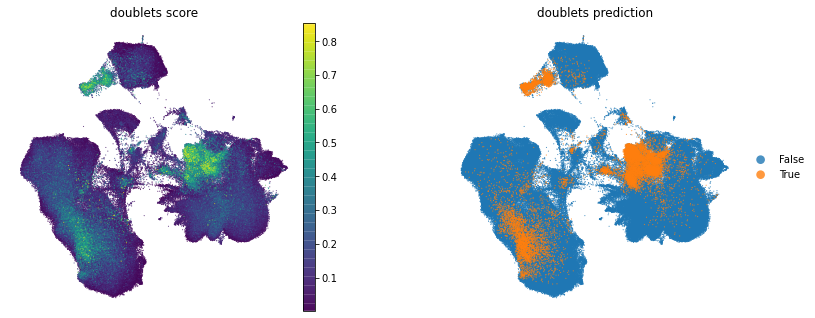


**Supplementary Figure 20. Doublets prediction results from Scrublet.**

# Supplementary Notes

## Cell-type annotation

In the lymphoid cell population, we define 5 CD8^+^ T cell subpopulations, including CD8n T cell, CD8m T(GZMK), CD8m T(GZMH), CD8m T(IL7R) and CD8 effector cells. The CD8n T cells cluster next to the CD4 naive T cells and express *CD8B, CD8A, CD3D, CD3E* and *CCR7*. CD8m T(GZMK), CD8m T(GZMH) and CD8m T(IL7R) are 3 subpopulations of the CD8 T cells and show complementary marker genes (*GZMK, GZMH* and *IL7R*), **Supplementary Figure 2**. *GZMH* is known as a cytotoxic effector T cell marker, while *GZMK* is a transitional effector T cell marker[^11^](https://paperpile.com/c/fq4a9u/OuaOa). *GZMH* and *GZMK* not only show good discrimination of the CD8^+^ T cell subpopulations, but also discriminates the NK cell populations, **Supplementary Figure 3**. The CD8eff T cell is a proliferating cell subpopulation and is easily distinguishable.

Given that γδ T cells comprise 1%–10% of human PBMCs, it is difficult to distinguish the γδ T cell subpopulations within a single dataset, moreover, the existing annotations of γδ T cells are not fully consistent: Wilk et al. 2020[^2^](https://paperpile.com/c/fq4a9u/0wrEn) annotated according to the γδ TCR constant chains encoding genes (*TRGC1, TRGC2* and *TRDC*), while Zhang et al. 2020[^3^](https://paperpile.com/c/fq4a9u/3KfYA) annotated γδ T cells as TRGV9^+^TRDV2^+^. However, these groups of marker genes highlight two cell subpopulations, **Supplementary Figure 4**. It is known that Vγ9Vδ2 T cells are the major subset of γδ T cells in human PBMCs[^12^](https://paperpile.com/c/fq4a9u/2SIHH). According to the marker knowledge of γδ T cells[^12–14^](https://paperpile.com/c/fq4a9u/2SIHH+bwSl4+2LDjX), we define the TRGV9^+^TRDV2^+^ subpopulation as Vγ9Vδ2 T cells and other CD161(KLRB1)^+^TRGC1^+^TRGC2^+^ cells as γδ T cells. The γδ T cells also express canonical γδ T cell markers *CCR5, CCR6*[*^15^*](https://paperpile.com/c/fq4a9u/DFTYL) as well as *SLC4A10*[*^16^*](https://paperpile.com/c/fq4a9u/mAGCl) and *TRAV1-2*[*^17^*](https://paperpile.com/c/fq4a9u/LWpBH), which are known as Mucosal-associated invariant T (MAIT) cell markers **Supplementary Figure 4**.

*CD56* (*NCAM1*), *GNLY* and *NKG7* highlight the NK cells. We further divide the NK cells into 4 subpopulations NK (GZMH^+^), NK(GZMK), NK(SYNE2) and mito-high NK. The mito-high NK cluster has a higher level of percentage of mitochondrial RNA contents and also expresses T cell markers (*CD3D, CD3E*) as well as some red blood cell markers (*HBB, HBA1, HBA2*). Therefore, the mito-high NK cluster is possibly a contaminated subpopulation.

We broadly divided the B cells into naïve B, memory B and plasma cells, where the naive B-cells were further divided into Naïve B(EEF1G), Naïve B(TMSB4X) and Naïve B(IGHD) based on the marker genes’ expression and memory B-cells were further divided into Memory B(EEF1G) and Memory B(CD27).

## Differentially expressed genes list

### 2.1 Genes downregulated in T cells, NK cells, DC, pDC, B cells, Plasma cells and Neutrophils:

*IFITM3*

*IFI6*

*IFI16*

*IFI44*

*IFI44L*

*IFIT3*

*ISG15*

*OAS1*

*OAS2*

*OAS3*

*OASL*

### 2.2 Genes downregulated in NK cells:

*MT-ATP6*

*MT-CO1*

*MT-CO2*

*MT-CO3*

*MT-CYB*

*MT-ND4*

*MT-ND5*

*MT-ND3*

*RPL13*

*MT-ND1*

*MT-ND2*

*RPL3*

*RPS2*

*RPS16*

*KLRB1*

*UBA52*

### 2.3 Genes downregulated in γδ T cells:

*MT-CO1*

*MT-CO2*

*MT-CO3*

*MT-CYB*

*MT-ATP6*

*RPL13*

*RPS18*

*MT-ND3*

*MT-ND4*

*RPL21*

*MT-ND2*

*UBA52*

*MT-ND1*

*RPS11*

*RPL23*

*RPL13A*

*KLRB1*

## Clonal expansion analysis of specific T-cell and B-cell subpopulations using T-cell receptor and B-cell receptor data respectively

### 3.1 T-cell subpopulation

Among the various T-cell subpopulations, we found no consistent trend for memory CD8^+^ T-cells (**Supplementary Figure 15d**) and memory CD4^+^ T-cells (**Supplementary Figure 16e**). An increased clonal expansion of γδ T-cells, with more than 5 clones in each clonotype was observed in recovered patients compared to healthy ones across all the three PBMC datasets (**Supplementary Figure 15d**). Naive CD8+ T-cells showed increased clonal expansion in severe patients in Zhang et al. 2020[^3^](https://paperpile.com/c/fq4a9u/3KfYA) compared to healthy control (group ‘Expnd (2<=n<=5)’) and similar clonal expansion status in convalescent patients compared to healthy controls in Zhang et al. 2020[^3^](https://paperpile.com/c/fq4a9u/3KfYA) (**Supplementary Figure 16c**). Whereas for naive CD4+ T-cells, the convalescent stage appeared to be similar to healthy across all the three PBMC datasets (**Supplementary Figure 16d**). Further, due to the presence of less than 100 CD8^+^ effector T-cells in most of the samples, we were unable to analyze this subpopulation in greater detail (**Supplementary Figure S16a-b**). We also observed that all the samples from the Liao et al. 2020[^7^](https://paperpile.com/c/fq4a9u/iNtel) TCR dataset had less than 100 cells each for most of the T-cell subpopulations (**Supplementary Figure S16a**), thereby leaving us with only one dataset, Zhang et al. 2020[^3^](https://paperpile.com/c/fq4a9u/3KfYA), with samples from severe and moderate stages.

### 3.2 B-cell subpopulation

Similar to CD8^+^ effector T-cells, there were less than 100 plasma cells with corresponding BCR data in most of the samples to draw any reliable conclusions in the per-study analysis (**Supplementary Figure 18b**). We observed slightly higher clonal expansion in naïve B-cells in convalescent COVID-19 patients compared to healthy controls in both the studies (clonally expanded clonotypes with 2 to 5 clones each; group ‘Expnd (2<=n<=5)’; **Supplementary Figure 18c**), but this difference was not statistically significant. We also observed increased clonal expansion of naïve B-cells in moderate and severe COVID-19 patients compared to healthy controls in both the clonally expanded groups (‘Expnd (2<=n<=5)’ and ‘Expnd (n>5); **Supplementary Figure 18c**) but as the Wen dataset lacks samples from these stages, this observation is not applicable to the Wen dataset. We didn’t observe any consistent trend in clonal expansion of memory B-cells across stages (**Supplementary Figure 18d**).

## Other possible discussion/limitations of the meta-analysis

We also checked whether samples at moderate stage from other datasets could be merged with samples at mild stage from Lee et al. 2020[^1^](https://paperpile.com/c/fq4a9u/SInZk) dataset, but these two stages exhibited different cell-proportion distributions (**Figure 3**) and differentially expressed genes and were, therefore, kept as separate. In addition, presence of more TCR/BCR data, longitudinal samples with a detailed history of received therapeutic interventions, particularly those with immunomodulatory effects such as Azithromycin[^18^](https://paperpile.com/c/fq4a9u/fFI49) given to severe patients in the Wilk dataset[^2^](https://paperpile.com/c/fq4a9u/0wrEn), additional clinical data on pre-existing conditions and ethnicities[^19^](https://paperpile.com/c/fq4a9u/EkpdD), comparison with asymptomatic patients and presence of other omics modalities[^20–24^](https://paperpile.com/c/fq4a9u/Mdmkw+11zDE+ke6vH+IFukS+htU0c) would have helped to strengthen the current analysis.

## Interactive visualization on cellxgene through usegalaxy.eu

The first time that you do this, you will need to go through all the steps. On subsequent visits you can just visit the link that you obtain on step 6. The interactive visualisation link will expire after 1 month possibly, but a new one can be generated again from step 4 and on.

If you don’t have an account at usegalaxy.eu, register for one at <https://usegalaxy.eu/login> and then login with that account.

Once logged:

1.- Go to <https://usegalaxy.eu/u/pmoreno/h/garg-et-al-2020-covid-19-single-cell-immune-response-meta-analysis> find the Galaxy history with the data.

2.- Press the import button (+) at the upper right corner next to “About this History”:


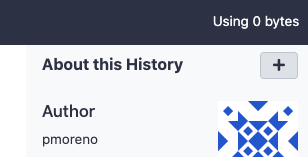


3.- Press Import in the dialog


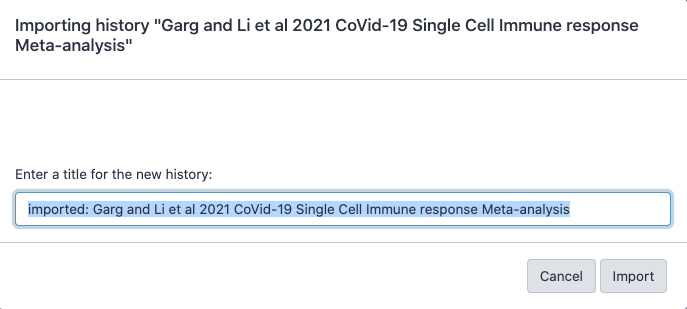


4.- Go to <https://usegalaxy.eu/root?tool_id=interactive_tool_cellxgene>

5.- Making sure that the field Concatenate Dataset that appear in the middle has this dataset


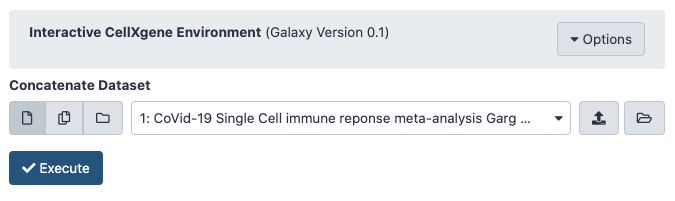


Click on Execute, you should see execution to the right on the history.

6.- The central page will show this at the top:


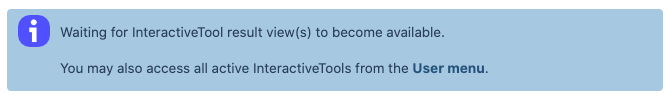


And then after a short while (this can take longer if the cluster is under heavy use) it should change to this:


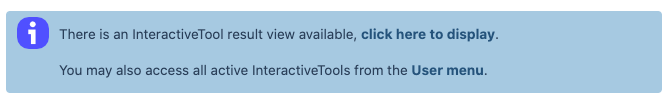


Click on “click here to display” and you should arrive to cellxgene with the merged dataset loaded. This link should be valid for at least 30 days, keep it so that you can go by directly to your cellxgene instance next time (if you have lost the link, there are indications to get it back after the final step).

7.- Enter a name on the text field to create an annotation collation to start exploring the dataset on cellxgene:


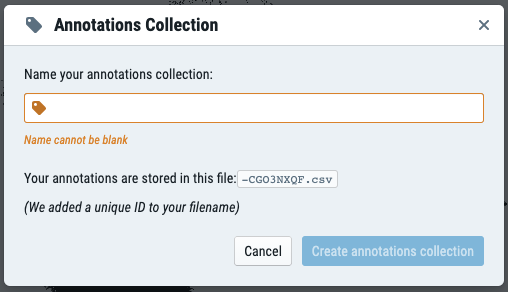


What if I didn’t keep the link to the cellxgene instance?

1.- Once you are logged in, go to the Active InteractiveTools item in the User menu.


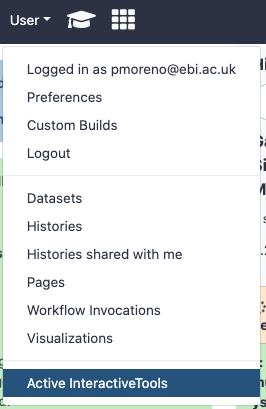


2.- You should see an entry like this, click on the name:


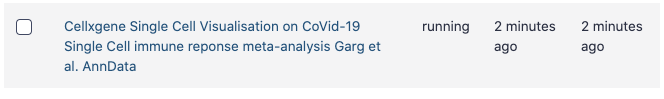


Clicking on it should take you to your own deployment of cellxgene with the dataset.

# References

1. [Lee, J. S. *et al.* Immunophenotyping of COVID-19 and influenza highlights the role of type I interferons in development of severe COVID-19. *Sci Immunol* **5**, (2020).](http://paperpile.com/b/fq4a9u/SInZk)

2. [Wilk, A. J. *et al.* A single-cell atlas of the peripheral immune response in patients with severe COVID-19. *Nat. Med.* **26**, 1070–1076 (2020).](http://paperpile.com/b/fq4a9u/0wrEn)

3. [Zhang, J.-Y. *et al.* Single-cell landscape of immunological responses in patients with COVID-19. *Nat. Immunol.* **21**, 1107–1118 (2020).](http://paperpile.com/b/fq4a9u/3KfYA)

4. [Wen, W. *et al.* Immune cell profiling of COVID-19 patients in the recovery stage by single-cell sequencing. *Cell Discov* **6**, 31 (2020).](http://paperpile.com/b/fq4a9u/fC7uS)

5. [Yu, K. *et al.* Thymosin alpha-1 protected T cells from excessive activation in severe COVID-19. *Research Square* (2020) doi:](http://paperpile.com/b/fq4a9u/mXGPk)[10.21203/rs.3.rs-25869/v2](http://dx.doi.org/10.21203/rs.3.rs-25869/v2)[.](http://paperpile.com/b/fq4a9u/mXGPk)

6. [Jiang, Q. Single cell and immune repertoire profiling of COVID-19 patients reveal novel therapeutic candidates. (2020) doi:](http://paperpile.com/b/fq4a9u/wCvws)[10.5281/zenodo.3747336](http://dx.doi.org/10.5281/zenodo.3747336)[.](http://paperpile.com/b/fq4a9u/wCvws)

7. [Liao, M. *et al.* Single-cell landscape of bronchoalveolar immune cells in patients with COVID-19. *Nature Medicine* vol. 26 842–844 (2020).](http://paperpile.com/b/fq4a9u/iNtel)

8. [He, J. *et al.* Single-cell analysis reveals bronchoalveolar epithelial dysfunction in COVID-19 patients. *Protein & cell* vol. 11 680–687 (2020).](http://paperpile.com/b/fq4a9u/CjM2F)

9. [Chua, R. L. *et al.* COVID-19 severity correlates with airway epithelium–immune cell interactions identified by single-cell analysis. *Nat. Biotechnol.* **38**, 970–979 (2020).](http://paperpile.com/b/fq4a9u/btvog)

10. [Ren, X. *et al.* COVID-19 immune features revealed by a large-scale single cell transcriptome atlas. *Cell* (2021) doi:](http://paperpile.com/b/fq4a9u/mqnAe)[10.1016/j.cell.2021.01.053](http://dx.doi.org/10.1016/j.cell.2021.01.053)[.](http://paperpile.com/b/fq4a9u/mqnAe)

11. [Li, H. *et al.* Dysfunctional CD8 T Cells Form a Proliferative, Dynamically Regulated Compartment within Human Melanoma. *Cell* vol. 181 747 (2020).](http://paperpile.com/b/fq4a9u/OuaOa)

12. [Künkele, K.-P. *et al.* Vγ9Vδ2 T Cells: Can We Re-Purpose a Potent Anti-Infection Mechanism for Cancer Therapy? *Cells* **9**, (2020).](http://paperpile.com/b/fq4a9u/2SIHH)

13. [Oliver Nussbaumer, M. T. Functional Phenotypes of Human Vγ9Vδ2 T Cells in Lymphoid Stress Surveillance. *Cells* **9**, (2020).](http://paperpile.com/b/fq4a9u/bwSl4)

14. [Schirmer, L., Rothhammer, V., Hemmer, B. & Korn, T. Enriched CD161high CCR6+ γδ T cells in the cerebrospinal fluid of patients with multiple sclerosis. *JAMA Neurol.* **70**, 345–351 (2013).](http://paperpile.com/b/fq4a9u/2LDjX)

15. [Glatzel, A. *et al.* Patterns of chemokine receptor expression on peripheral blood gamma delta T lymphocytes: strong expression of CCR5 is a selective feature of V delta 2/V gamma 9 gamma delta T cells. *J. Immunol.* **168**, (2002).](http://paperpile.com/b/fq4a9u/DFTYL)

16. [Park, D. *et al.* Differences in the molecular signatures of mucosal-associated invariant T cells and conventional T cells. *Sci. Rep.* **9**, (2019).](http://paperpile.com/b/fq4a9u/mAGCl)

17. [Wong, E. B. *et al.* TRAV1-2 + CD8 + T-cells including oligoconal expansions of MAIT cells are enriched in the airways in human tuberculosis. *Communications Biology* **2**, 1–13 (2019).](http://paperpile.com/b/fq4a9u/LWpBH)

18. [Lin, S.-J., Kuo, M.-L., Hsiao, H.-S. & Lee, P.-T. Azithromycin modulates immune response of human monocyte-derived dendritic cells and CD4+ T cells. *Int. Immunopharmacol.* **40**, 318–326 (2016).](http://paperpile.com/b/fq4a9u/fFI49)

19. [Wong, W. S. *et al.* Reference ranges for lymphocyte subsets among healthy Hong Kong Chinese adults by single-platform flow cytometry. *Clin. Vaccine Immunol.* **20**, 602–606 (2013).](http://paperpile.com/b/fq4a9u/EkpdD)

20. [Su, Y. *et al.* Multi-Omics Resolves a Sharp Disease-State Shift between Mild and Moderate COVID-19. *Cell* **183**, 1479–1495.e20 (2020).](http://paperpile.com/b/fq4a9u/Mdmkw)

21. [Overmyer, K. A. *et al.* Large-Scale Multi-omic Analysis of COVID-19 Severity. *Cell Syst* **12**, 23–40.e7 (2021).](http://paperpile.com/b/fq4a9u/11zDE)

22. [Bernardes, J. P. *et al.* Longitudinal Multi-omics Analyses Identify Responses of Megakaryocytes, Erythroid Cells, and Plasmablasts as Hallmarks of Severe COVID-19. *Immunity* **53**, 1296–1314.e9 (2020).](http://paperpile.com/b/fq4a9u/ke6vH)

23. [Barh, D. *et al.* Multi-omics-based identification of SARS-CoV-2 infection biology and candidate drugs against COVID-19. *Comput. Biol. Med.* **126**, 104051 (2020).](http://paperpile.com/b/fq4a9u/IFukS)

24. [Stephenson, E. *et al.* The cellular immune response to COVID-19 deciphered by single cell multi-omics across three UK centres. *bioRxiv* (2021) doi:](http://paperpile.com/b/fq4a9u/htU0c)[10.1101/2021.01.13.21249725](http://dx.doi.org/10.1101/2021.01.13.21249725)[.](http://paperpile.com/b/fq4a9u/htU0c)
